# Fronto-parietal oscillatory dynamics of emotion regulation as a function of adult attachment orientations

**DOI:** 10.3389/fnhum.2026.1742378

**Published:** 2026-05-07

**Authors:** Marcos Domic-Siede, Javiera Figueroa-Cuevas, Krishna Leiva-Cortés, Daniela López, Mónica Guzmán-González, Thomas Lau-Lemus, Sara Hernández, Romina Ortiz, Lesly González, Jaime R. Silva

**Affiliations:** 1 Escuela de Psicología, Universidad Católica del Norte, Antofagasta, Chile; 2 EmoPsy Lab, Faculty of Psychology, Universidad del Desarrollo, Santiago, Chile; 3 Fundación Arturo López Pérez OECI Cancer Center, Unidad de Investigación Epidemiológica y Clínica, Centro de Investigación e Innovación en Cáncer, Santiago, Chile

**Keywords:** attachment theory, cognitive reappraisal, EEG source reconstruction, emotion regulation, expressive suppression, oscillatory dynamics

## Abstract

**Introduction:**

Emotion regulation enables individuals to modulate emotional experiences and behaviors according to situational demands. Within attachment theory, individual differences in attachment anxiety and avoidance are conceived as interpersonal dispositions that influence the quality and efficiency of emotion regulation strategies, potentially shaping the underlying neural dynamics. This study examined cortical oscillatory activity during two regulation strategies—cognitive reappraisal and expressive suppression—focusing on theta (4–8 Hz) and beta (15–30 Hz) bands at the source level.

**Methods:**

Forty adults (21 male, 18 female, 1 gender unspecified participant; *M* = 27.58 years, *SD* = 8.71) performed an emotion regulation task involving emotionally evocative images from the International Affective Picture System (IAPS) while EEG was recorded. Cortical sources were reconstructed using standardized low-resolution brain electromagnetic tomography (sLORETA). Linear mixed-effects models (LMMs) assessed the effects of condition, region of interest (ROI), and attachment orientations on oscillatory power.

**Results:**

Results showed that higher attachment anxiety predicted reduced theta power in the right dorsolateral prefrontal cortex (dlPFC) during reappraisal, indicating attenuated recruitment of cognitive control mechanisms. In the beta band, suppression reduced activity in the right parietal lobe/precuneus—a region involved in self-referential and attentional processes—across participants, while during reappraisal, attachment anxiety was linked to lower beta power and attachment avoidance to higher beta power in the left dlPFC.

**Discussion:**

Together, these findings indicate that cognitive reappraisal and expressive suppression engage distinct cortical oscillatory systems, and that interpersonal dispositions modulate their neural implementation. Frontal theta appears to index top-down control during reappraisal, whereas beta activity shows a dual pattern: frontal beta variations reflect differences in control stability associated with attachment orientations, and parietal beta decreases during suppression suggest attentional disengagement from self-referential processing.

## 1 Introduction

### 1.1 Attachment theory

Attachment theory describes an innate motivational system that promotes proximity-seeking behavior toward a caregiver under threat. This proximity provides safety and supports emotion regulation (Bowlby, 1969, 1973, 1982; Mikulincer and Shaver, 2016). Through early interactions, individuals develop internal working models of the self and others that shape expectations about relationships (Bowlby, 1973; Brennan et al., 1998). In adulthood, attachment is commonly conceptualized along two dimensions: anxiety and avoidance (Brennan et al., 1998; Mikulincer and Shaver, 2016; Guzmán-González et al., 2020). Attachment anxiety involves a negative view of the self and a fear of rejection or abandonment, leading to hyperactivating strategies such as clinging and heightened emotional expression. Attachment avoidance reflects a negative view of others and discomfort with intimacy, promoting deactivating strategies such as emotional suppression and withdrawal (Mikulincer and Shaver, 2003; Bartholomew and Horowitz, 1991; Hazan and Shaver, 1987). Secure attachment (low anxiety and avoidance) is characterized by positive expectations about self and others, supporting more adaptive emotional responses (Mikulincer and Shaver, 2016; Messina et al., 2024). In contrast, attachment insecurity—whether anxious or avoidant—is consistently linked to emotion regulation difficulties and maladaptive affective responses (Cassidy and Shaver, 2016; Guzmán-González et al., 2016; Han and Kahn, 2017; Henschel et al., 2020; Long et al., 2020; Vrtička and Vuilleumier, 2012).

### 1.2 Emotion regulation

Emotion regulation refers to processes that shape which emotions people experience, when they occur, and how they are expressed (Gross, 1998, 2002). In Gross’s process model (Gross, 1998; Olderbak et al., 2023), two key families of strategies are cognitive change and response modulation. Cognitive change includes cognitive reappraisal, which involves reframing an emotion-eliciting situation to alter its emotional impact (Gross, 2002; Uusberg et al., 2014). Response modulation includes expressive suppression, or the conscious inhibition of emotional expressions (Gross, 2002; Goldin et al., 2008; Olderbak et al., 2023). Strategies differ in adaptiveness. Reappraisal is generally associated with higher well-being and better social functioning, whereas suppression is often linked to greater distress and poorer relational outcomes (Gross and John, 2003; Goldin et al., 2008; Olderbak et al., 2023).

Attachment orientations strongly shape both the selection and effectiveness of regulation strategies. Individuals with higher attachment security tend to use reappraisal more effectively and flexibly, whereas attachment anxiety and avoidance are associated with maladaptive regulation patterns (Mikulincer and Shaver, 2016; Guzmán-González et al., 2016; Long et al., 2020; Ramos-Henderson et al., 2024; Domic-Siede et al., 2024; Vrtička and Vuilleumier, 2012). Anxiously attached individuals often show emotional hyperactivation, rumination, and reduced control (Henschel et al., 2020; Ramos-Henderson et al., 2024), while avoidantly attached individuals tend to rely on suppression and show reduced efficacy in reappraisal (Vrtička and Vuilleumier, 2012).

Emotion regulation is not only cognitive but also socially embedded. Early relational experiences shape expectations about others’ availability and responsiveness, which in turn influence how people use strategies such as reappraisal and suppression (Mikulincer and Shaver, 2016). From this perspective, the ability to regulate emotion develops in the context of interpersonal relationships, particularly attachment bonds, which guide how individuals manage affect in social contexts throughout life.

### 1.3 Neural bases of emotion regulation

Emotion regulation is implemented by a set of interconnected neural systems involving prefrontal, limbic, and parietal regions. Neuroimaging studies, primarily using functional magnetic resonance imaging (fMRI), have consistently implicated the prefrontal cortex (PFC) as a critical region for top-down regulation of emotional responses, particularly in down-regulating activity in emotion-generative structures like the amygdala (Buhle et al., 2014; Morawetz et al., 2017; Ochsner et al., 2002, 2012). Within the PFC, different subregions have specialized functions. The dorsolateral prefrontal cortex (dlPFC) is central for cognitive control and working memory, supporting the manipulation of information required for reappraisal (Buhle et al., 2014). The ventrolateral prefrontal cortex (vlPFC) contributes to the selection and inhibition of responses, particularly in relation to suppressing inappropriate emotional behaviors (Morawetz et al., 2017). The dorsomedial prefrontal cortex (dmPFC) and anterior cingulate cortex (ACC) are implicated in conflict monitoring and self-referential processing, playing a role in evaluating the success of regulatory efforts (Etkin et al., 2015; Ochsner et al., 2012). Finally, parietal regions, including the inferior parietal lobule and precuneus, contribute to attentional reorienting and visuospatial processing during regulation. According to classic cortical organization models (Northoff et al., 2006), the precuneus—part of the medial cortical system—also supports self-referential processing, linking internal representations of the self with ongoing emotional and cognitive states (Ochsner et al., 2012).

The amygdala, a key limbic structure, is reliably activated during emotional experience and is commonly targeted by regulation processes. Successful reappraisal is associated with decreased amygdala activation, which reflects reduced emotional reactivity (Buhle et al., 2014; Goldin et al., 2008). Conversely, when individuals engage in suppression or when regulation fails, amygdala activity often remains high or increases (Ochsner et al., 2012). These findings support the idea that reappraisal is more efficient than suppression in terms of attenuating the neural correlates of negative affect.

The PFC–amygdala interaction is a core circuit for emotion regulation: PFC regions implement cognitive control that can down-regulate amygdala responses to emotional stimuli (Etkin et al., 2015). Connectivity studies suggest that stronger inverse coupling between the PFC and amygdala (i.e., greater prefrontal control over limbic reactivity) predicts better regulation outcomes and lower affective symptoms (Morawetz et al., 2017; Ochsner et al., 2012). This frontolimbic model has informed much of the contemporary understanding of emotion regulation at the neural level.

Critically, evidence linking attachment orientations to frontolimbic circuitry is mixed. Some studies report that secure attachment is associated with more efficient prefrontal down-regulation of amygdala responses during emotional challenges (Vrtička et al., 2008, 2012; Vrtička and Vuilleumier, 2012). A meta-analysis found the most consistent differences in lateral PFC activity (often reduced in avoidance) and amygdala activity (often elevated in anxiety) (Ran and Zhang, 2018). However, findings vary across paradigms and task demands (Long et al., 2020). Anxious attachment has been linked to heightened limbic responsivity and inefficient prefrontal recruitment, reflecting increased sensitivity to social threat and difficulty down-regulating distress, whereas avoidant attachment often shows blunted amygdala reactivity together with attenuated or disengaged lateral prefrontal activity, consistent with deactivating or suppression-based strategies. Overall, the data point to context-dependent modulations within frontolimbic networks, emphasizing variability in how attachment dispositions shape emotional control at the neural level.

Electrophysiological measures have also complemented fMRI findings by providing fine-grained temporal information about emotion regulation processes. Event-related potentials (ERPs), such as the late positive potential (LPP), have been widely used to index sustained attentional engagement with emotional stimuli and its modulation during regulation (Hajcak and Nieuwenhuis, 2006; Moser et al., 2006). ERP studies, including some examining individual differences in attachment, have shown that emotion regulation can influence the temporal dynamics of emotional processing (e.g., Ramos-Henderson et al., 2024; Domic-Siede et al., 2025a).

Together, fMRI and EEG studies indicate that the neural bases of emotion regulation are deeply intertwined with attachment orientations, influencing both the effectiveness and preferred strategies of emotional control. Building on this literature, the present work leverages EEG to focus on oscillatory dynamics as candidate markers of the fronto-parietal implementation of regulation and its modulation by attachment orientations.

### 1.4 Brain oscillations

Electroencephalography (EEG) provides a powerful tool for examining the temporal dynamics of neural processes underlying emotion regulation. Within EEG, oscillatory measures complement time-locked ERP components by capturing sustained rhythmic coordination that supports distributed control processes during regulation. EEG oscillatory activity—i.e., rhythmic patterns in neuronal firing—reflects different modes of neural communication and support cognitive and affective functions (Başar et al., 2001; Buzsáki and Draguhn, 2004; Klimesch, 1999). Among the various frequency bands, theta (4–8 Hz) and beta (13–30 Hz) oscillations have been most consistently implicated in emotion regulation (Abid et al., 2025; Domic-Siede et al., 2024, 2025b; Knyazev, 2007; Peng et al., 2025). Frontal midline theta activity is commonly associated with cognitive control and mental effort, especially in tasks requiring conflict resolution, working memory, or sustained attention (Cavanagh and Frank, 2014). During emotion regulation tasks, increases in theta power have been interpreted as reflecting the engagement of top-down regulatory processes, such as reappraisal (Ertl et al., 2013). Theta synchronization is believed to support the functional integration of distributed cortical and subcortical systems (Cavanagh and Frank, 2014), facilitating the implementation of regulatory strategies in real time. Beta activity, by contrast, has been associated with both motor inhibition and the maintenance of cognitive and emotional states (Engel and Fries, 2010; Heinrichs-Graham and Wilson, 2016; Gilbertson et al., 2005). In the context of emotion regulation, beta oscillations—particularly over frontal and parietal regions—are thought to reflect the effortful inhibition of emotional expression (Davidson et al., 2000; Domic-Siede et al., 2025b). Thus, theta and beta oscillations provide complementary indices of regulatory engagement: theta signaling controlled modulation of emotion, and beta reflecting the inhibition of emotional expression.

Importantly, individual differences in attachment orientations may influence the pattern of oscillatory activity during regulation. Preliminary findings suggest that individuals higher in attachment anxiety exhibit decreased frontal theta activity and a reduced frontal theta synchrony during emotional tasks, consistent with increased regulatory effort or emotional reactivity (Domic-Siede et al., 2024, 2025b). Individuals higher in attachment avoidance, in turn, may show altered beta patterns, possibly reflecting habitual suppression or disengagement from emotional stimuli (Domic-Siede et al., 2025b). These findings offer a promising avenue for linking attachment theory to the neurophysiology of emotion regulation. Despite these advances, existing EEG findings linking attachment orientations to oscillatory activity have been derived primarily from channel-level analyses, which offer limited anatomical specificity. As a result, it remains unclear how distinct emotion regulation strategies—such as cognitive reappraisal and expressive suppression—are implemented at the level of cortical oscillatory sources within fronto-parietal networks, and how these source-level mechanisms vary as a function of attachment anxiety and avoidance.

### 1.5 The present study

The present study aimed to investigate the cortical oscillatory dynamics underlying two emotion regulation strategies—cognitive reappraisal and expressive suppression—and their modulation by attachment orientations. Specifically, we focused on theta (4–8 Hz) and beta (15–30 Hz) frequency bands at the source level to explore the neural mechanisms associated with cognitive control and expressive suppression, respectively. Our primary outcomes were source-level theta and beta power in fronto-parietal ROIs, and how these measures were modulated by condition and attachment orientations. The integration of attachment theory into the study of oscillatory brain activity represents an emerging direction in affective neuroscience—one that bridges individual differences in socio-emotional motivation with neurophysiological mechanisms of regulation (Long et al., 2020; Vrtička and Vuilleumier, 2012).

Individuals with higher attachment anxiety often exhibit hyperactivating responses—heightened emotional reactivity and difficulties engaging prefrontal control—whereas those with higher attachment avoidance tend to engage in deactivating responses, such as emotional suppression and withdrawal from affective cues (Long et al., 2020; White et al., 2020). These tendencies suggest distinct neural signatures that may be observable in oscillatory activity.

At the neural level, we expected frontal theta activity to index top-down control during cognitive reappraisal (Cavanagh and Frank, 2014; Ertl et al., 2013) and beta oscillations to reflect inhibitory control during expressive suppression (Engel and Fries, 2010; Heinrichs-Graham and Wilson, 2016). We therefore hypothesized that:

1. Higher attachment anxiety would predict reduced frontal theta during reappraisal, indicating less efficient prefrontal engagement.
2. Higher attachment avoidance would predict increased beta power during suppression, consistent with enhanced motor inhibition and emotional disengagement.

To test these hypotheses, we used source-level EEG analyses based on a multi-atlas anatomical framework (Desikan et al., 2006; Destrieux et al., 2010; Klein and Tourville, 2012). This approach allowed us to estimate oscillatory activity within well-defined cortical regions involved in emotion regulation, including the anterior cingulate cortex (ACC), medial and dorsolateral prefrontal cortices (mPFC, dlPFC), ventrolateral prefrontal cortex/inferior frontal gyrus (vlPFC/IFG), supplementary and pre-supplementary motor areas (SMA/pre-SMA; BA 6), and parietal cortex/precuneus (PL/Precuneus).

By combining attachment theory with electrophysiological evidence, this study sought to elucidate how interpersonal motivational systems modulate neural oscillatory mechanisms of emotion regulation. Such findings may contribute to an integrative understanding of the social and neurophysiological architecture of emotional control, offering novel insights into the interplay between attachment, cognition, and affect at the cortical level.

## 2 Materials and methods

### 2.1 Participants

A purposive, non-probabilistic sampling strategy was used to recruit adult participants (≥18 years old) with normal or corrected-to-normal vision and no history of severe neuropsychiatric disorders. All participants provided written informed consent prior to participation. Recruitment was conducted through digital platforms of the School of Psychology at Universidad Católica del Norte, Chile.

The initial sample comprised 46 participants. Six were excluded due to excessive EEG noise or technical issues, resulting in a final sample of 40 adults (38 Chilean, 1 Argentinian, and 1 Bolivian): 18 females (*M* = 27.44 years, SD = 8.33; range = 19–52), 21 males (*M* = 27.81 years, SD = 9.12; range = 19–57), and one participant with unspecified sex/gender (age = 26). The overall sample had a mean age of *M* = 27.58 years (SD = 8.71; range = 19–57) (Table 1).

**Table 1:** Sociodemographic characteristics of the participants (*n* = 40).

Variables	*n*	%	Mean (SD)
Age			27.58 (8.60)
Sex			
Male	21	52.50	
Female	18	45.00	
Other	1	2.50	
Nationality			
Chilean	38	95.00	
Bolivian	1	2.50	
Argentine	1	2.50	
Marital status			
Single	33	82.50	
Married	7	17.50	
Socioeconomic level			
Low	1	2.50	
Low–Middle	21	52.50	
Upper–Middle	18	45.00	
High	0	0.00	
Education			
Completed Secondary Education	3	7.50	
Completed Technical Education	4	10.00	
Completed Higher Education	9	22.50	
Incomplete Technical/Higher Education	20	50.00	
Postgraduate studies	4	10.00	
Occupation			
Employed	20	50.00	
Homemaker	2	5.00	
Technical/Higher Education Student	18	45.00	

An *a priori* power analysis was conducted using G*Power 3.1.9.2 (Faul et al., 2007) for a repeated-measures ANOVA with three within-subject conditions (*α* = 0.05, power = 0.85, assumed correlation among measures = 0.50, nonsphericity correction = 1). The analysis indicated that a minimum of 31 participants was required to detect a medium effect size (*f* = 0.25). The final sample of 40 participants exceeded this threshold, ensuring sufficient statistical power for detecting effects of interest. To assess sensitivity, a post-hoc power analysis considered the significant interactions from our linear mixed-effects models: (i) attachment anxiety × reappraisal in the theta band at right dlPFC (*β* ≈ −0.19), and (ii) during reappraisal in the beta band at left dlPFC, attachment anxiety and avoidance showed opposite associations (*β* = −0.13 and *β* = +0.12, respectively). Given the achieved sample size (*N* = 40), α = 0.05, and a repeated-measures ANOVA framework with three within-subject conditions, the design was sensitive to effects of *f* = 0.22 (η^2^p = 0.05), consistent with small-to-medium effects typically observed in EEG emotion studies (e.g., Uusberg et al., 2014).

Ethical approval was granted by the Scientific Ethics Committee of Universidad Católica del Norte (Resolutions No. 099/2021 and 037/2023). All procedures adhered to the Declaration of Helsinki and institutional ethical guidelines for human research.

### 2.2 Instruments

#### 2.2.1 Experiences in close relationships questionnaire (ECR-12)

Attachment was evaluated using the Spanish adaptation of the 12-item Experiences in Close Relationships Questionnaire (ECR-12), validated for the Chilean population by Guzmán-González et al. (2020). This self-report measure assesses two dimensions of adult attachment: anxiety and avoidance, each represented by six items (e.g., “I feel uncomfortable opening up to my partner” for avoidance; “If I cannot get my partner to show interest in me, I get upset or angry” for anxiety) (Brennan et al., 1998). Participants responded on a 7-point Likert scale ranging from 1 (strongly disagree) to 7 (strongly agree), with higher scores reflecting greater attachment insecurity (Guzmán-González et al., 2023). In the present sample, the scale demonstrated satisfactory internal consistency (α = 0.76 for attachment anxiety, α = 0.84 for avoidance), aligning with previous psychometric findings for both the 12- and 36-item Chilean versions (Guzmán-González et al., 2020; Spencer et al., 2013).

### 2.3 Emotion regulation task

Emotion regulation was evaluated using an experimental paradigm adapted from Ochsner et al. (2004), Vrtička et al. (2012), and recent studies by Domic-Siede et al. (2024, 2025a, 2025b, 2025c). The task was implemented in *Presentation®* software (Version 18.0, Neurobehavioral Systems) and included 60 images from the International Affective Picture System (IAPS; Lang et al., 2005): 45 negative and 15 neutral, selected according to previous ER research (Moser et al., 2009; Schlumpf et al., 2019; Domic-Siede et al., 2023a) (see Supplementary Tables S1–S3).

Picture sets were selected based on IAPS normative valence and arousal ratings, following established procedures for this emotion regulation paradigm. Negative images were assigned to regulation conditions using a nested design, such that each image set was consistently paired with a single instructional condition (Natural-neutral, Natural-negative, Reappraise, or Suppress). To verify comparability across experimental conditions, differences between picture sets were statistically evaluated using normative IAPS ratings for individual images.

Given deviations from normality in valence ratings, a non-parametric Kruskal–Wallis test revealed a significant effect of condition (H = 35.43, *p* < 0.0001). *Post hoc* Dunn’s tests confirmed that the neutral picture set differed significantly from all negative conditions (Natural-negative, Reappraise, Suppress; all *p* < 0.001), whereas no significant differences were observed among the three negative conditions (Supplementary Tables S1–S3). Differences in normative arousal were analyzed using a one-way ANOVA after verifying normality with the Shapiro–Wilk test. The omnibus test was significant (*F* = 46.66, *p* < 0.0001), reflecting expected differences between neutral and negative images. Crucially, *post hoc* Tukey HSD comparisons indicated no significant differences in arousal among the negative image sets assigned to the Natural-negative, Reappraise, and Suppress conditions (see Supplementary Tables S1–S3). These analyses indicate that the negative picture sets were closely matched in normative valence and arousal.

Participants performed three experimental conditions—Natural, Reappraise, and Suppress (Domic-Siede et al., 2023a)—while stimuli were displayed on a 23.6-inch ASUS VG248QE monitor. Prior to the main task, a training phase was conducted consisting of three blocks of three practice trials per condition, which included visual examples and instructions for using the Self-Assessment Manikin (SAM; Bradley and Lang, 1994) to rate emotional arousal.

In the Natural condition, participants simply viewed each image and allowed emotions to occur spontaneously. In Reappraise, they employed cognitive reappraisal strategies to reinterpret the image (e.g., imagining a positive or fictional outcome; Ochsner et al., 2004). In Suppress, they were instructed to inhibit any facial or bodily expression of emotion. Following each image, participants rated the intensity of their emotional experience on a 7-point SAM scale (1 = low, 7 = high) in response to the prompt: “Indicate the intensity of your emotional response to the image you just saw.” Although the SAM allows assessment of both valence and arousal, the present study measured exclusively on arousal ratings. All emotional stimuli used in the regulation conditions were selected to be highly negatively valenced based on normative IAPS ratings, thereby restricting variability in stimulus valence by design. Arousal was expected to vary as a function of the regulation strategy applied and thus provided a more sensitive index of emotional intensity. To minimize task burden and fatigue, participants provided a single, brief rating after each trial.

The task comprised 12 randomized blocks of five trials each. The Natural condition included 30 trials (15 neutral and 15 negative: Nat-neutral and Nat-negative), while Reappraise and Suppress each included 15 trials using only negative images. Each participant viewed the same fixed image set, with every image assigned exclusively to one condition (Natural, Reappraise, or Suppress). Both the block order and the trial sequence within blocks were randomized individually for each participant. Short breaks were provided between blocks to reduce fatigue. This design was adopted to avoid stimulus-specific carryover effects that may arise when the same emotional images are repeatedly associated with different regulation instructions within a single experiment. Although this approach limits full stimulus counterbalancing, it preserves the interpretability of regulation contrasts by minimizing strategy-related carryover effects.

**Figure 1:**
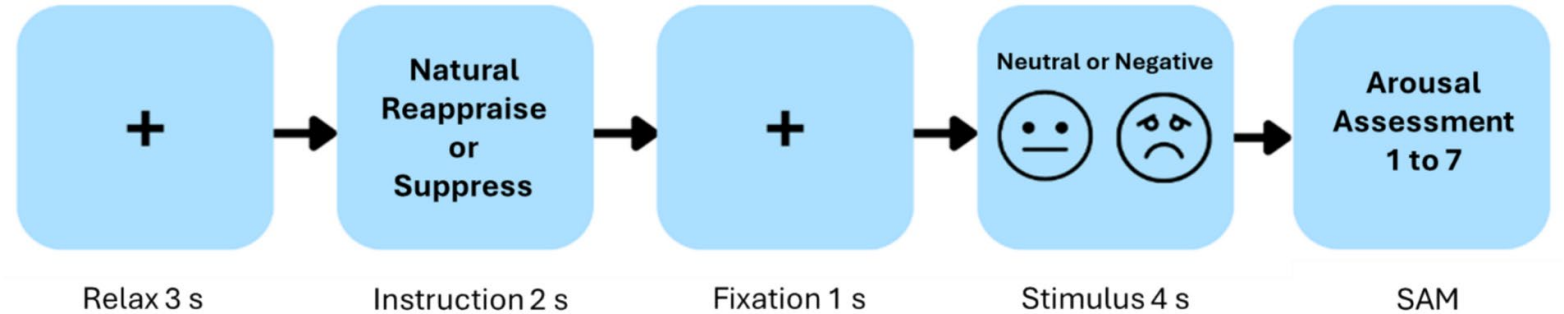
The diagram depicts the sequence of events within a representative trial of the experimental task used to examine how different emotion regulation strategies influence affective responses. Each trial starts with a 3-s relaxation period indicated by a fixation cross, followed by a 2-s cue instructing participants to apply one of three strategies: respond naturally, suppress their emotional expression, or reappraise the meaning of the upcoming stimulus. After a 1-s fixation interval, a neutral or negative image is presented for 4 s. Immediately afterward, participants report their perceived arousal on a 7-point Likert scale, where 1 denotes low emotional intensity and 7 denotes high emotional intensity. SAM, Self-Assessment Manikin.

Each trial followed a fixed temporal sequence (Figure 1): (i) a 3-s fixation cross, (ii) a 2-s instruction cue, (iii) a 1-s fixation with variable duration (1000–1,100 ms, jittered across trials), (iv) a 4-s image presentation, and (v) the arousal rating screen. This sequence was repeated across all trials in all conditions. The jittered pre-stimulus fixation interval was included to reduce temporal predictability and minimize anticipatory neural activity (e.g., CNV-like buildup) prior to image onset.

### 2.4 Electroencephalographic data acquisition

Electroencephalographic (EEG) activity was recorded using a BioSemiⓇ system^1^ equipped with 64 scalp electrodes and two mastoid electrodes, arranged according to the international 10/20 system (Keil et al., 2014). The signal was continuously sampled at 2048 Hz and referenced online to the Common Mode Sense (CMS) and Driven Right Leg (DRL) active electrodes. All electrode impedances were maintained below 20 kΩ throughout the recording.

### 2.5 Data analysis

#### 2.5.1 Behavioral data analysis

Behavioral data were processed using *GraphPad Prism 8* and *MATLAB R2022b*. Descriptive statistics were computed to summarize participants’ self-reported arousal ratings. Data normality was evaluated with the Shapiro–Wilk test (Mishra et al., 2019), which indicated significant deviations and skewness; therefore, non-parametric analyses were employed.

To verify that the experimental manipulation produced the expected changes in emotional arousal, a Friedman test was conducted as the non-parametric equivalent of a repeated-measures ANOVA (Field, 2013). When significant main effects were observed, Dunn’s post-hoc comparisons were applied, and Cohen’s *d* was computed to estimate effect sizes for each pairwise contrast.

This analysis served as a manipulation check to confirm the overall task effect on arousal across conditions.

In addition, to evaluate whether stimulus-specific variability could bias the observed behavioral effects due to the nested stimulus–condition design, an additional trial-level linear mixed-effects analysis was conducted in MATLAB. In this model, self-reported arousal ratings were predicted by Condition as a fixed effect, while including random intercepts for both Subject and Stimulus (nested within Condition). This approach allowed us to explicitly model stimulus-level variance and assess whether the observed differences between conditions could be attributed to a subset of specific images rather than to the experimental manipulation itself.

Model comparisons were performed to determine whether including stimulus as a random effect significantly improved model fit, and whether the main effect of Condition remained significant after accounting for stimulus-level variability. This analysis served as a robustness check to ensure that behavioral effects were not driven by stimulus-specific confounds inherent to the fixed stimulus–condition mapping. This approach follows recent recommendations emphasizing the importance of treating stimuli as random factors in experimental designs involving repeated exposure to stimulus sets (e.g., Bauer et al., 2020; Judd et al., 2012).

#### 2.5.2 EEG signal preprocessing

EEG data were preprocessed following established procedures (Domic-Siede et al., 2021, 2023b, 2024, 2025c) using EEGLAB toolbox (Delorme and Makeig, 2004). First, a high-pass finite impulse response (FIR) filter at 0.1 Hz was applied to remove slow drifts, followed by a low-pass filter at 40 Hz to attenuate high-frequency noise. The continuous data were then downsampled to 512 Hz.

Epochs were extracted from −1,000 ms to 4,000 ms relative to the onset of the IAPS image for four conditions: Natural Neutral, Natural Negative, Reappraise, and Suppress. Trials containing gross artifacts were visually excluded. Artifact correction was further refined through Independent Component Analysis (ICA) using the Logistic Infomax algorithm (Bell and Sejnowski, 1994). Components corresponding to non-neural sources (e.g., eye blinks, muscle activity, cardiac artifacts) were identified with ICLabel (Pion-Tonachini et al., 2019) and verified by visual inspection. This preprocessing pipeline maximized the signal-to-noise ratio while maintaining spatial accuracy for subsequent analyses.

After artifact rejection, the number of retained trials per condition was examined to ensure sufficient data for subsequent analyses. On average, participants contributed a comparable number of trials across conditions (Natural Negative: *M* = 14.45, SD = 0.96; Neutral: *M* = 14.40, SD = 1.06; Reappraise: *M* = 14.35, SD = 1.41; Suppress: *M* = 14.75, SD = 0.59). The minimum number of trials per condition ranged from 9 to 13, indicating that the vast majority of trials were retained following preprocessing. These values indicate a relatively balanced number of trials across conditions after artifact rejection, supporting the reliability of the estimated neural measures.

#### 2.5.3 Brain sources reconstruction

Cortical source estimation was performed using the open-access software Brainstorm (Tadel et al., 2011), available under the GNU General Public License.^2^ Source reconstruction was applied to preprocessed EEG data (filtered between 0.1 and 40 Hz and artifact-free) corresponding to the 4-s image-viewing period in each trial. The sources were estimated using the Standardized Low-Resolution Brain Electromagnetic Tomography (sLORETA) algorithm (Pascual-Marqui, 2002), based on the ICBM152 anatomical template and the standard 10–20 electrode system. The minimum-norm imaging approach was implemented using the symmetric Boundary Element Method (BEM) forward model provided by OpenMEEG (Gramfort et al., 2010).

##### 2.5.3.1 Theta band source (4–8 Hz)

Oscillatory power in the theta band was estimated directly from the reconstructed cortical sources. For each participant and condition, the power spectral density (PSD) was computed using the Welch method (1-s Hamming windows with 50% overlap). The PSD was calculated separately for the task period (0.3–4 s post-stimulus) and for the baseline period (−1 to 0 s). The 0.3–4 s window was selected *a priori* based on prior EEG studies of emotion regulation showing that regulatory engagement and sustained affective processing occur within this interval (e.g., Domic-Siede et al., 2024, 2025a, 2025b; Ertl et al., 2013; Hajcak and Nieuwenhuis, 2006; Moser et al., 2006). Theta power during the task was then normalized using a symmetric contrast between the task and baseline periods, defined as:


$$\text{Normalized power}=\frac{A-B}{A+B}$$

where A corresponds to the PSD during the task period and B to the PSD during the baseline period. This baseline normalization controls for tonic pre-stimulus activity and inter-trial physiological variability.

**Figure 2:**
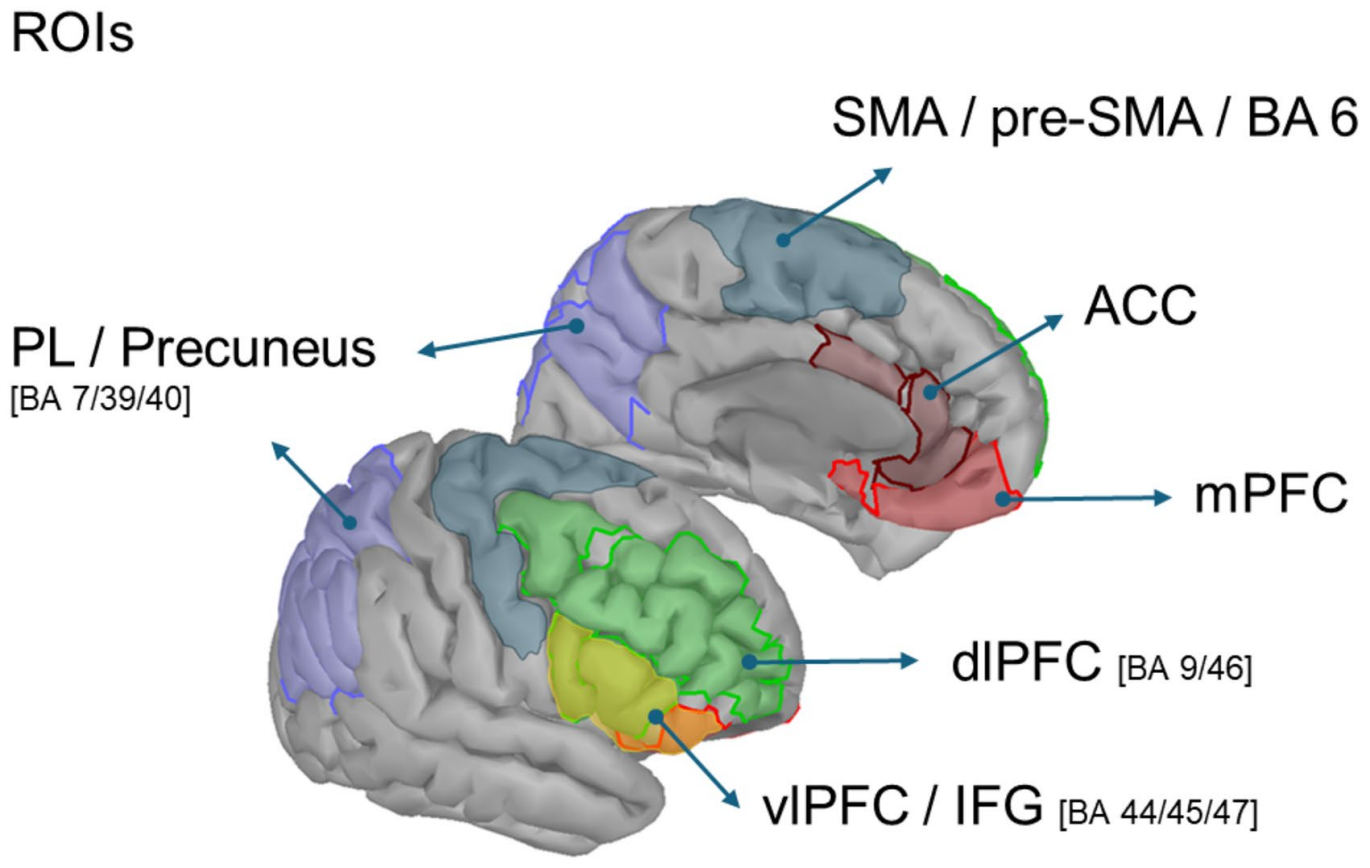
Regions of interest (ROIs) selected for source-level analysis. Cortical regions of interest were defined according to the Destrieux atlas and bilateral Brodmann areas. ROIs included the anterior cingulate cortex (ACC), medial prefrontal cortex (mPFC), dorsolateral prefrontal cortex (dlPFC; BA 9/46), ventrolateral prefrontal cortex/inferior frontal gyrus (vlPFC/IFG; BA 44/45/47), supplementary and pre-supplementary motor areas (SMA/pre-SMA; BA 6), and parietal lateral cortex/precuneus (PL/Precuneus; BA 7/39/40). These regions were used to extract principal component time series (PCA) from the reconstructed source activity within the theta (4–8 Hz) and beta (15–30 Hz) frequency bands.

Regions of interest (ROIs) were anatomically defined using a multi-atlas convergence approach based on the Destrieux atlas (Destrieux et al., 2010), Brodmann areas (Fischl, 2012), the Desikan–Killiany–Tourville (DKT) atlas (Klein and Tourville, 2012), and the Desikan–Killiany atlas (Desikan et al., 2006). The following bilateral regions were selected: anterior cingulate cortex (ACC), dorsolateral prefrontal cortex (dlPFC), medial prefrontal cortex (mPFC), and ventrolateral prefrontal cortex/inferior frontal gyrus (vlPFC/IFG). For each ROI, a representative time series was extracted using Principal Component Analysis (PCA), and the first principal component was selected as the index of dominant theta activity (Figure 2).

##### 2.5.3.2 Beta band source (15–30 Hz)

The analysis of beta oscillatory activity followed the same procedure used for the theta band. PSD was computed with the Welch method in the 15–30 Hz range for both the task (0.3–4 s) and baseline (−1 to 0 s) periods. This temporal window was also defined a priori, consistent with previous work on emotion regulation (Domic-Siede et al., 2024, 2025a, 2025b; Ertl et al., 2013; Hajcak and Nieuwenhuis, 2006; Moser et al., 2006). Normalized beta power was obtained using the same symmetric contrast (A − B)/(A + B), with A corresponding to task PSD and B to baseline PSD. This baseline normalization controls for tonic pre-stimulus activity and inter-trial physiological variability.

The bilateral ROIs defined for beta analysis (derived from the same multi-atlas framework: Destrieux, Brodmann, DKT, and Desikan atlases) included the supplementary motor and premotor area (BA6), parietal lateral cortex / precuneus (PL/Precuneus), dorsolateral prefrontal cortex (dlPFC), and ventrolateral prefrontal cortex / inferior frontal gyrus (vlPFC/IFG). As in the theta analysis, PCA was applied within each ROI, and the first component was used as the representative measure of dominant beta activity. Normalized PSD maps were averaged across participants for each condition. Group-level cortical maps were spatially normalized and projected onto the ICBM152 template surface for visualization. Cortical activations were displayed using Brainstorm’s default colormap and threshold settings, illustrating the spatial distribution of task-related power changes in the theta and beta frequency bands.

#### 2.5.4 Linear mixed-effects models

To test the hypotheses regarding the modulation of oscillatory power by Condition (Negative, Reappraise, Suppress), Region of Interest (ROI), and affective covariates (Attachment Anxiety [ANX] and Attachment Avoidance [AVD]), we fitted linear mixed-effects models (LMMs) separately for the theta and beta frequency bands using MATLAB (fitlme, maximum likelihood estimation).

##### 2.5.4.1 Data preprocessing and coding

- Subject was treated as a random factor.
- Condition had three levels (Negative, Reappraise, Suppress), with Negative as the reference.
- ROI was modeled as a categorical fixed factor, with different sets for each frequency band:

 o Theta band: L/R ACC, L/R dlPFC, L/R mPFC, L/R vlPFC/IFG (reference = *Left vlPFC/IFG*).
 o Beta band: L/R BA6, L/R PL/Precuneus, L/R dlPFC, L/R vlPFC/IFG (reference = *Left BA6*).

- Continuous covariates (ANX and AVD) were mean-centered at the sample level: ANX_c, AVD_c.
- The dependent variable was Amplitude (log-transformed, normalized oscillatory power).

##### 2.5.4.2 Fixed-effects structure

Each model included all main effects and their interactions up to three-way terms among Condition, ROI, and the covariates:


$$\text{mplitude}∼\underset{\text{task and topography effects}}{\underset{︸}{\text{Condition}\times \mathrm{ROI}}}\times ({\mathrm{ANX}}_{c}+{\mathrm{AVD}}_{c})$$

This expands to:

- Main effects: Condition, ROI, ANX_c, AVD_c
- Two-way interactions: Condition×ROI, Condition×ANX_c, ROI × ANX_c, Condition×AVD_c, ROI × AVD_c
- Three-way interactions: Condition×ROI × ANX_c and Condition×ROI × AVD_c

This structure allowed us to test whether Reappraise increased theta activity in frontal ROIs, and whether higher attachment anxiety attenuated this effect (Condition×ROI × ANX_c), as well as analogous modulations by AVD.

##### 2.5.4.3 Random-effects structure

To account for inter-individual variability, we specified random intercepts and slopes for Condition by subject, as well as ROI-specific intercepts nested within each subject:


(1+Condition∣Subject)+(1∣Subject:ROI)

This structure models (a) subject-level variation in both intercepts and condition slopes (and their correlations), and (b) ROI-level variability within subjects.

##### 2.5.4.4 Inference and reporting

- Degrees of freedom and *p*-values were estimated using the Satterthwaite approximation (default in fitlme).
- Fixed effects were reported as *β* (SE), *t*, *p*, and 95% confidence intervals.
- Model fit indices included AIC, BIC, and log-likelihood values.
- Simple slopes and planned contrasts (e.g., effect of ANX_c within *Reappraise × R dlPFC*) were tested using coefTest, with marginal predictions (predict) at ±1 SD of ANX_c or AVD_c.

##### 2.5.4.5 Robustness and influence diagnostics

Residuals, leverage values, and Cook’s distance were examined for influential subjects or ROI-level observations. We also verified the stability of key effects (e.g., *Condition×ROI×ANX_c* in the right dlPFC, theta band) through leave-one-subject-out analyses. Main results are reported along with these checks when they affected inference.

#### 2.5.5 Spearman rank correlations

To complement the mixed-effects models, we conducted non-parametric Spearman rank correlations to examine bivariate associations among behavioral and neural variables. These analyses were designed to assess the functional significance of the cortical sources identified in the LMMs as showing the strongest task- and trait-related effects. Specifically, only the right dlPFC (theta band) and left dlPFC (beta band) were selected, as these regions and frequency bands exhibited significant or theoretically meaningful modulations by condition and attachment orientations in the multilevel analyses (see Section 3.3).

Beyond their neural interpretation, these correlations also served to explore whether individual differences in attachment orientations were reflected in subjective emotional experience. This approach provided a complementary behavioral index testing whether anxious and avoidant attachments were associated with differential emotional reactivity and regulation success across conditions. This targeted analysis therefore allowed us to determine (a) whether the magnitude of source activity covaried with subjective arousal, and (b) whether attachment-related traits mapped onto individual differences in emotional intensity across emotion regulation contexts.

##### 2.5.5.1 Neural–behavioral correlations

(1) Right dlPFC theta (4–8 Hz) × arousal: Following the *a priori* effect tested in the LMMs, we correlated normalized theta power from the right dlPFC ROI with self-reported arousal separately for each condition (Neutral, Reappraise, Suppress, Negative).
(2) Left dlPFC beta (15–30 Hz) × arousal: Motivated by the left frontal beta effects in the LMMs, we correlated normalized beta power from the left dlPFC ROI with arousal separately for each condition.

*Behavior–trait correlations*: We also examined the association between self-reported arousal and attachment dimensions (ECR-12 Anxiety [ANX], Avoidance [AVD]) within each condition to test whether individual differences in attachment mapped onto perceived emotional intensity.

## 3 Results

### 3.1 Behavioral results

Self-reported arousal ratings (1–7 scale) were compared across four conditions: Natural Neutral, Reappraise, Suppress, and Natural Negative. Descriptive analyses revealed a clear progressive increase in arousal from Natural Neutral (*M* = 1.79, SD = 0.97) to Natural Negative (*M* = 3.51, SD = 1.37), with intermediate values for Reappraise (*M* = 2.49, SD = 1.05) and Suppress (*M* = 2.86, SD = 1.42) (Supplementary Table S4). Although the Shapiro–Wilk test indicated that Reappraise data did not deviate from normality (*p* = 0.099), other conditions showed non-normal distributions (*ps* < 0.05; Supplementary Table S5). Therefore, a nonparametric Friedman test was applied, revealing a significant main effect of Condition, χ^2^(3) = 57.34, *p* < 0.0001 (Supplementary Table S6).

**Figure 3:**
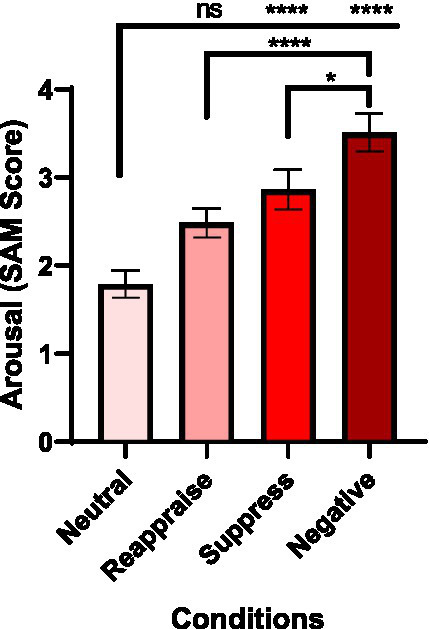
Self-reported arousal across emotion regulation conditions. Mean Self-Assessment Manikin (SAM) arousal ratings (1–7 scale) are shown for the four experimental conditions: Neutral, Reappraise, Suppress, and Negative. A Friedman test revealed a significant main effect of Condition, χ^2^(3) = 57.34, *p* < 0.0001. Post-hoc Dunn’s tests indicated higher arousal in Suppress (*p* < 0.0001, d = 0.97) and Negative (*p* < 0.0001, *d* = 1.57) relative to Neutral. Negative images also elicited greater arousal than Reappraise (*p* < 0.0001, *d* = 1.00) and Suppress (*p* = 0.038, d = 0.60). Error bars represent ±1 SEM. Significance levels: *p < 0.05; ****p < 0.0001; ns, not significant.

Post-hoc Dunn’s multiple comparisons (Supplementary Tables S7, S8) indicated significantly higher arousal in Suppress (*p* < 0.0001, *d* = 0.97) and Natural Negative (*p* < 0.0001, *d* = 1.57) compared to Natural Neutral. The Reappraise condition did not differ significantly from Neutral (*p* = 0.0638), nor from Suppress (*p* = 0.4138). However, Reappraise and Suppress both elicited lower arousal than Natural Negative (*p*s < 0.0001, *d*s = 1.00 and 0.60, respectively; Figure 3).

These results indicate that the Suppress and Natural Negative conditions produced markedly greater perceived arousal relative to the neutral baseline. In contrast, although Reappraise tended to increase arousal compared to Neutral, its levels remained lower than those in the Natural Negative condition, consistent with its regulatory effectiveness. Although these differences are consistent with a regulatory effect, the present design does not allow us to fully determine whether variation in the distribution of normative arousal values within each stimulus set contributed to the magnitude of these effects. These analyses served as a manipulation check to confirm the expected condition effect on arousal.

To further examine whether the observed behavioral effects could be influenced by stimulus-specific variability inherent to the nested stimulus–condition design, we conducted an additional trial-level linear mixed-effects analysis. In this model, arousal ratings were predicted by Condition as a fixed effect, with random intercepts specified for both Subject and Stimulus nested within Condition.

Model comparison indicated that including stimulus as a random factor significantly improved model fit relative to a model including only subject-level variability (ΔAIC = 71.5; likelihood ratio test *p* < 0.001), confirming the presence of stimulus-related variability in self-reported arousal. However, critically, the main effect of Condition remained statistically significant after accounting for this stimulus-level variance (Reappraise: *β* = −0.535, SE = 0.145, *p* = 0.0002; Suppress: *β* = −0.284, SE = 0.144, *p* = 0.0498; Neutral: *β* = −1.207, SE = 0.145, *p* < 0.0001; reference = Natural Negative).

Inspection of variance components further indicated that the variability attributable to stimuli (SD = 0.34) was substantially smaller than between-subject variability (SD = 1.28), suggesting that stimulus-specific differences contributed only modestly to overall variance in arousal responses. Additionally, descriptive analyses at the stimulus level showed no evidence that a small subset of images disproportionately drove the observed condition effects.

Together, these findings suggest that, although stimulus-related variability is present, observed behavioral differences across conditions are unlikely to be explained solely by stimulus-specific effects. This analysis provides additional support for the robustness of the experimental manipulation at the behavioral level.

### 3.2 Brain sources reconstruction results

#### 3.2.1 Global source reconstruction in theta band (4–8 Hz) – *Reappraise* condition

The cortical distribution of theta power (4–8 Hz) during the Reappraise condition showed clear positive contrasts (A−B)/(A+B) in frontal and midline regions compared with baseline (−1 to 0 s). The strongest theta activity appeared in the right dorsolateral prefrontal cortex (dlPFC), right ventrolateral prefrontal cortex/inferior frontal gyrus (vlPFC/IFG), and right medial prefrontal cortex (mPFC), with additional increases in posterior parietal cortex and precuneus.

**Figure 4:**
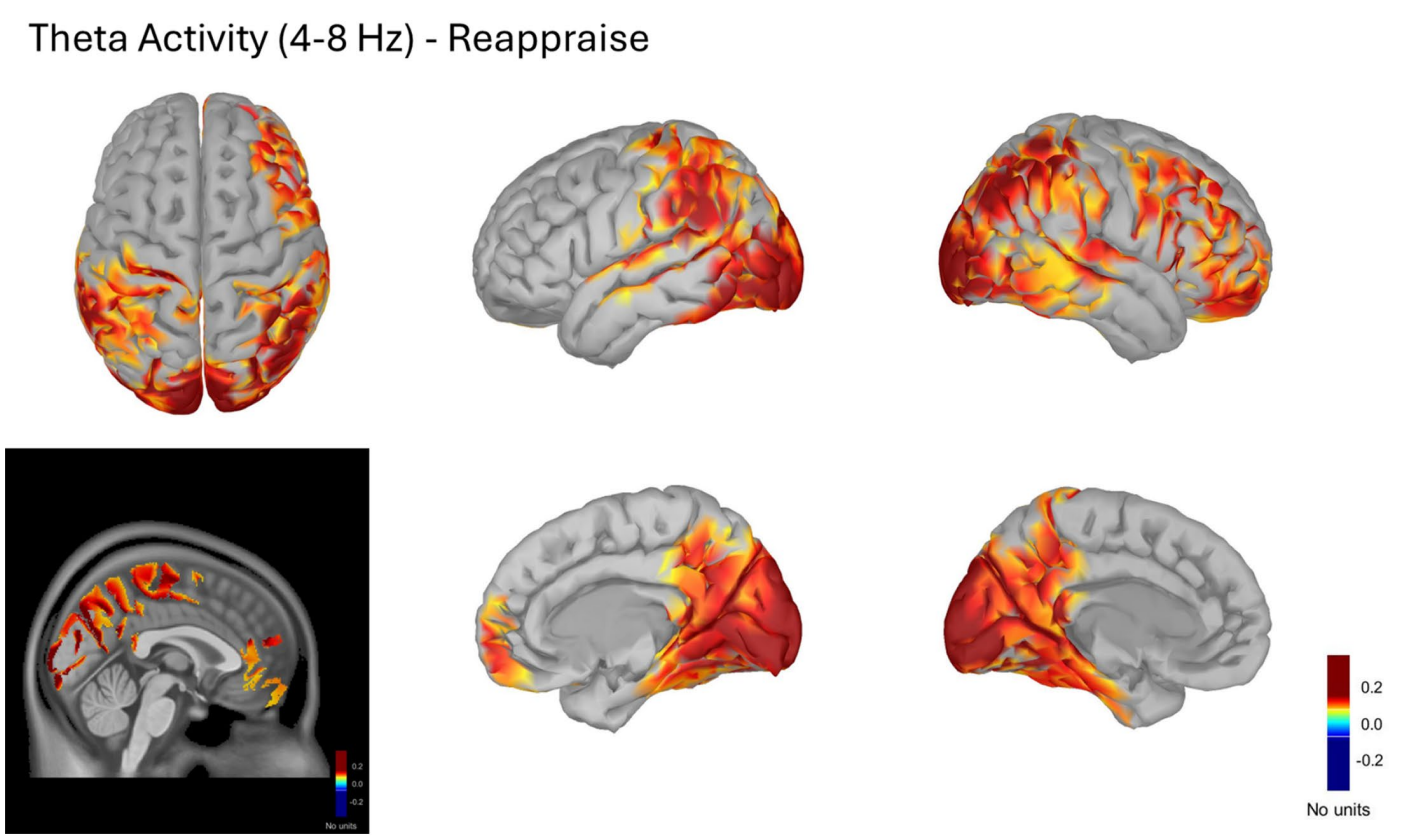
Cortical theta (4–8 Hz) source activity during the Reappraise condition. Normalized power spectral density (PSD) maps computed using the (A, B)/(A + B) contrast between task (A) and baseline (B) periods. PSD values for A and B were obtained with the Welch method in physical units of μV^2^/Hz; the normalization yielded unitless ratios representing relative changes in spectral power. The figure displays averaged theta activity from 0.3 to 4 s post-stimulus, projected onto the ICBM152 cortical surface. Warmer colors indicate increased theta power relative to baseline, predominantly in the right dorsolateral prefrontal cortex (dlPFC), ventrolateral prefrontal cortex/inferior frontal gyrus (vlPFC/IFG), medial prefrontal cortex (mPFC), and posterior parietal regions including the precuneus.

This spatial pattern indicates predominant fronto-posterior engagement consistent with the recruitment of top-down control networks typically linked to cognitive reappraisal. Figure 4 shows the normalized theta source maps projected on the ICBM152 template for the 0.3–4 s post-stimulus interval.

#### 3.2.2 Global source reconstruction in beta band (15–30 Hz) – *Suppress* condition

The Suppress condition displayed a more heterogeneous modulation of beta power (15–30 Hz) relative to baseline, with both positive and negative contrasts across the cortex. Decreases in beta activity (blue-coded) were visible in portions of the left dlPFC (BA 9/46), right vlPFC/IFG (BA 44/45), bilateral SMA/pre-SMA, bilateral mPFC, and parts of the posterior parietal cortex. Conversely, increases (warm-coded) were observed over the bilateral precuneus (BA 7/39/40), posterior parietal and occipital cortices, bilateral mPFC, and right dlPFC.

**Figure 5:**
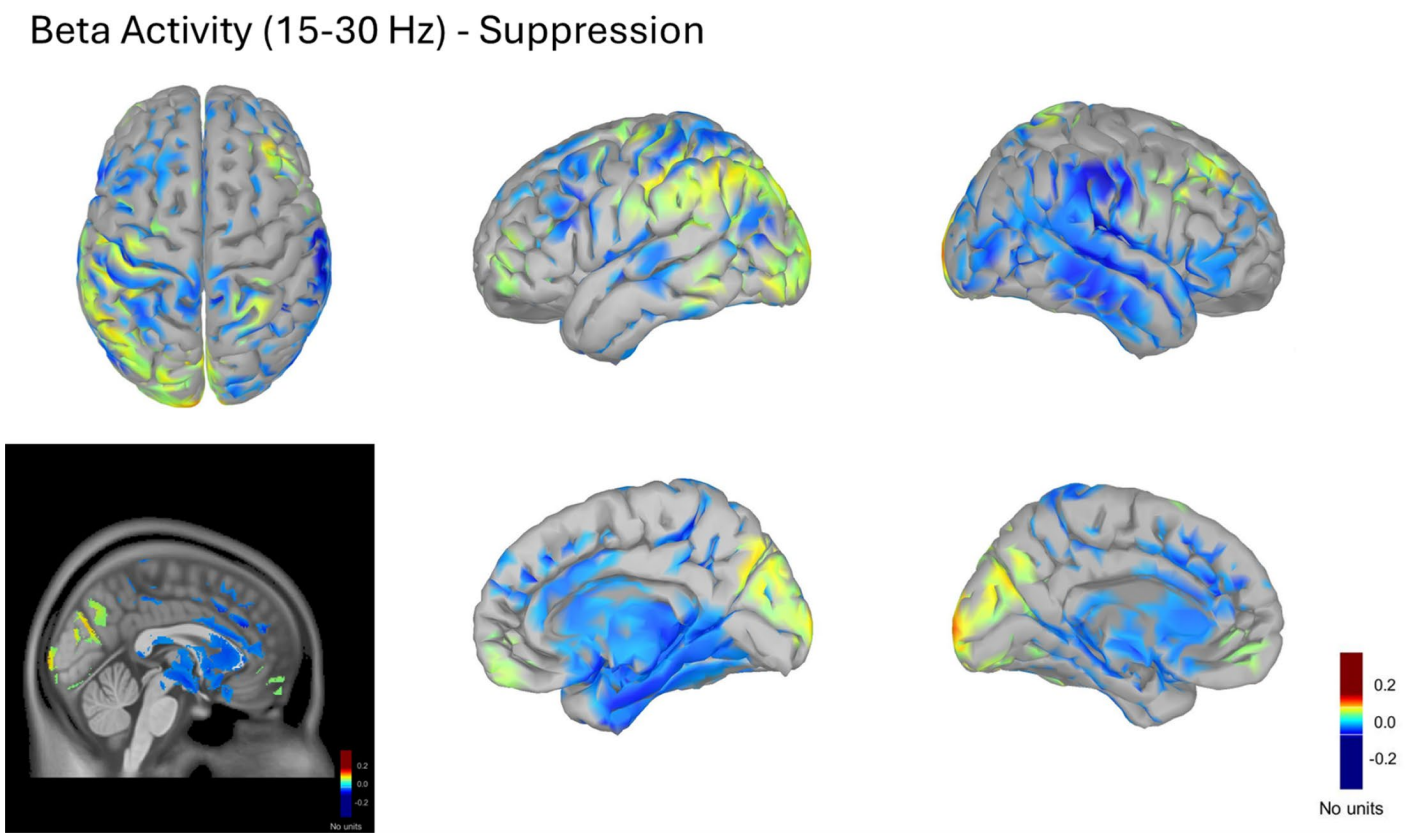
Cortical beta (15–30 Hz) source activity during the suppress condition. Normalized power spectral density (PSD) maps computed using the (A, B)/(A + B) contrast between task (A) and baseline (B) periods. PSD values for A and B were estimated with the Welch method in physical units of μV^2^/Hz; the normalization produced unitless ratios reflecting relative changes in spectral power. The figure displays averaged beta activity from 0.3 to 4 s post-stimulus, projected onto the ICBM152 cortical surface. Cooler colors (negative contrasts) indicate decreased beta power relative to baseline, particularly across posterior parietal cortex, precuneus, and medial frontal regions, whereas warmer colors (positive contrasts) mark localized increases in posterior and dorsolateral prefrontal areas.

This mixed configuration suggests differentiated fronto-parietal engagement: posterior and prefrontal beta enhancements possibly supporting higher-order control and self-monitoring, together with central beta decreases associated with reduced motor readiness during expressive suppression. Figure 5 depicts these normalized beta maps across multiple cortical views within the 0.3–4 s window.

### 3.3 Linear mixed-effects models results

We fit separate LMMs for theta and beta power (see Methods for structure). Both models converged under ML and showed acceptable residual dispersion.

#### 3.3.1 Model fit

The theta model (reference: Negative × L vlPFC/IFG) yielded AIC = 492.09, BIC = 881.44, log-likelihood = −166.04. The beta model (reference: Negative × L BA6) yielded AIC = −1.27, BIC = 388.09, log-likelihood = 80.64 (Table 2).

**Table 2:** Model fit indices.

Band	Reference	*N* (obs)	Fixed k	Random k	AIC	BIC	LogLik
Theta	Negative × L vlPFC/IFG	960	72	440	492.09	881.44	−166.04
Beta	Negative × L BA6	960	72	440	−1.27	388.09	80.64

#### 3.3.2 Theta band

There were no omnibus main effects of Condition across ROIs (ps > 0.23). Critically, we observed a Condition × ROI × ANX interaction specific to the right dlPFC during Reappraise: higher attachment anxiety predicted lower theta power (*β* = −0.187, SE = 0.075, *t* = −2.48, *p* = 0.013). This pattern is consistent with the hypothesis that anxiety attenuates frontal theta engagement during cognitive reappraisal (Table 3; Supplementary Tables S9, S10).

**Table 3:** Theta LMM — effects relevant to hypotheses.

Effect (theta)	*β*	SE	*t*	*p*
Condition: Reappraise	0.024	0.066	0.37	0.714
Condition: Suppress	0.082	0.069	1.20	0.232
ROI: R dlPFC	0.107	0.059	1.81	0.071
Reappraise × R dlPFC × ANX	**−0.187**	**0.075**	**−2.48**	**0.013**
Suppress × L mPFC × AVD	0.139	0.069	2.00	**0.046**
Suppress × R mPFC × AVD	0.140	0.070	2.09	**0.045**
Reappraise × ROI × AVD (any frontal)	—	—	—	≥0.19
Reappraise × ROI × ANX (others)	—	—	—	≥0.15

Values in bold indicate statistically significant effects (*p* < 0.05).

Attachment avoidance (AVD) did not significantly modulate theta during Reappraise (ps ≥ 0.19), but showed a *trend toward increased theta* during Suppress in the mPFC bilaterally (L mPFC: *β* = 0.139, SE = 0.069, *t* = 2.00, *p* = 0.046; R mPFC: *β* = 0.140, SE = 0.069, *t* = 2.01, *p* = 0.045). The right dlPFC also exhibited a trend toward higher theta overall (*β* = 0.107, SE = 0.059, *p* = 0.071) (Table 2; Supplementary Tables S9, S10).

Random effects indicated substantial between-subject variability in condition slopes (Reappraise SD = 0.197; Suppress SD = 0.232) with negative intercept–slope correlations (see Supplementary Table S11).

#### 3.3.3 Beta band

No omnibus Condition main effects emerged (ps ≥ 0.32). Task × ROI effects showed lower beta during Suppress in right PL/Precuneus (*β* = −0.216, SE = 0.067, *t* = −3.25, *p* = 0.001, Supplementary Figure S1), alongside a positive main effect for right PL/Precuneus (*β* = 0.113, SE = 0.048, *p* = 0.018) (Table 3; Supplementary Table S12).

Affective covariates modulated beta selectively in left dlPFC during Reappraise: ANX was associated with lower beta (*β* = −0.127, SE = 0.061, *t* = −2.08, *p* = 0.038), whereas AVD was associated with higher beta (*β* = 0.124, SE = 0.056, *t* = 2.21, *p* = 0.027). No other ANX/AVD interactions reached significance (ps ≥ 0.08) (Table 4; Supplementary Table S13).

**Table 4:** Beta LMM — effects relevant to hypotheses.

Effect (beta)	*β*	SE	*t*	*p*
Condition: Reappraise	0.050	0.050	1.00	0.315
Condition: Suppress	0.034	0.048	0.70	0.483
ROI: R PL/Precuneus	0.113	0.048	2.38	0.018
Suppress × R PL/Precuneus	**−0.216**	**0.067**	**−3.25**	**0.001**
Reappraise × L dlPFC × ANX	**−0.127**	**0.061**	**−2.08**	**0.038**
Reappraise × L dlPFC × AVD	**0.124**	**0.056**	**2.21**	**0.027**

Values in bold indicate statistically significant effects (*p* < 0.05).

In summary, reappraisal-related frontal theta was attenuated by higher attachment anxiety in the right dlPFC, whereas attachment avoidance showed only a trend-level increase in theta during suppression in the mPFC. For beta power, suppression decreased beta in the right parietal lobe and precuneus, while during reappraisal, attachment anxiety decreased and attachment avoidance increased beta in the left dlPFC.

### 3.4 Correlations between dlPFC theta power and self-reported arousal

**Figure 6:**
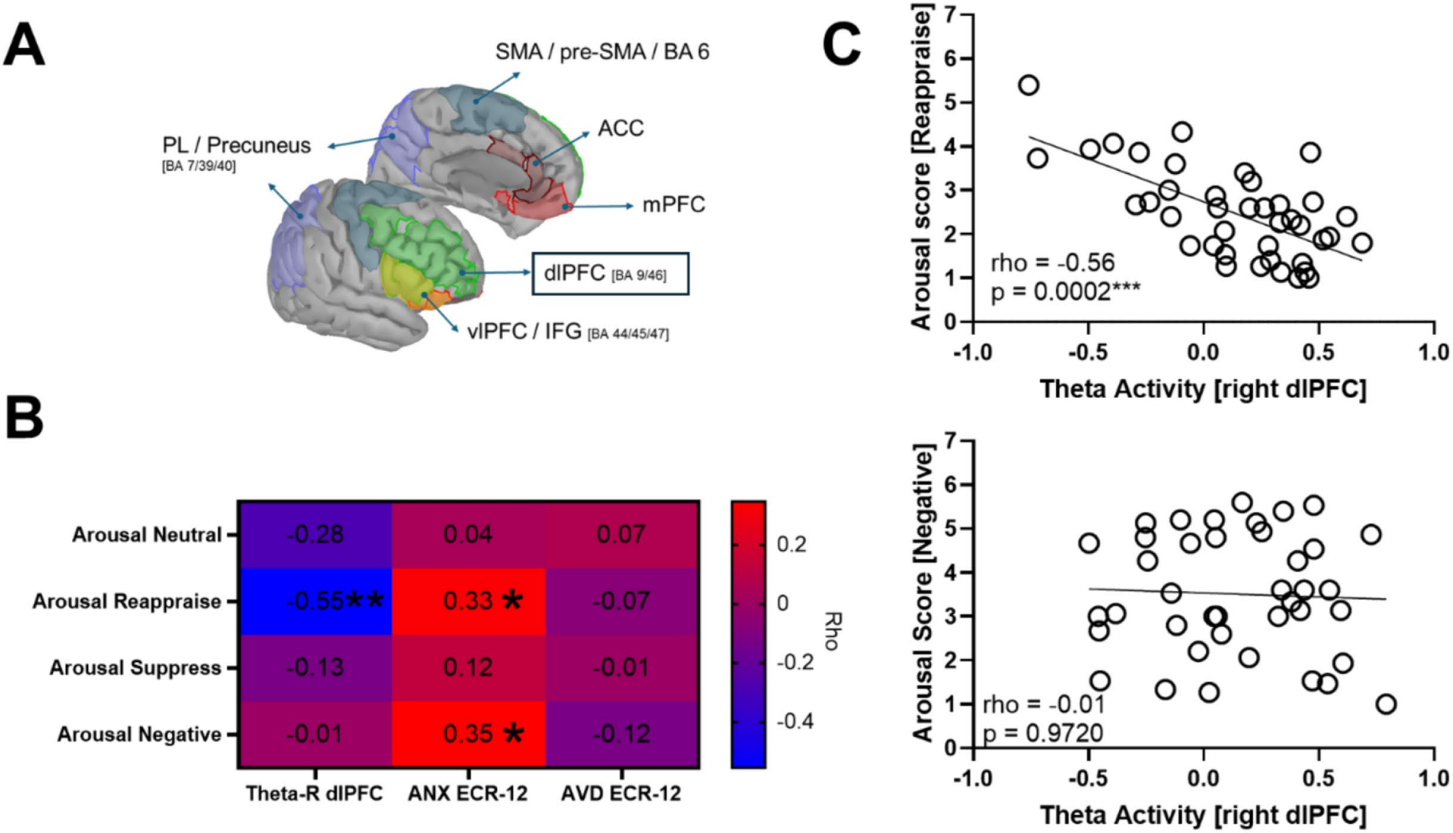
Associations between right dlPFC theta activity, self-reported arousal, and attachment orientations. **(A)** Cortical localization of the right dorsolateral prefrontal cortex (R dlPFC) region of interest (ROI) used for correlation analyses, displayed on the ICBM152 cortical template. **(B)** Heatmap showing Spearman’s rho coefficients between theta power in the R dlPFC (first column), attachment anxiety (ANX; second column), and attachment avoidance (AVD; third column) with self-reported arousal across the four experimental conditions (rows). Blue hues denote negative correlations and red hues positive correlations; *p* < 0.05 (*), *p< 0.01 (**). **(C)** Scatter plots illustrating the negative association between R dlPFC theta power and arousal during the Reappraise condition (top) and the absence of correlation during the Negative condition (bottom).

To further examine the functional relevance of the right dorsolateral prefrontal cortex (R dlPFC) theta activity observed during the Reappraise condition (Figure 6A), we tested whether self-reported arousal levels in each condition were associated with theta power extracted from this ROI. In addition, these analyses provided an opportunity to examine whether subjective emotional responses varied as a function of attachment dimensions, serving as a behavioral correlate of the neural effects and offering indirect evidence that individuals with different attachment orientations experience and regulate emotions differently.

Spearman correlations revealed a significant negative association between theta power in the R dlPFC and self-reported arousal during the Reappraise condition (rho = −0.55, *p* = 0.0002), indicating that greater frontal theta activity was linked to lower perceived arousal while reappraising negative stimuli (Figures 6B,C, top). No significant correlations emerged between theta and arousal in the Neutral (rho = −0.28, *p* = 0.076), Suppress (rho = −0.13, *p* = 0.435), or Negative (rho = −0.01, *p* = 0.972) conditions (Figures 6B,C, bottom).

Regarding attachment dimensions, attachment anxiety correlated positively with arousal during both Reappraise (rho = 0.33, *p* = 0.036) and Negative (rho = 0.35, *p* = 0.028) conditions, suggesting that individuals with higher attachment anxiety reported greater emotional activation in response to negative stimuli and during attempts to regulate them. In contrast, no significant associations were found between arousal and attachment avoidance across any condition (all ps > 0.44).

### 3.5 Correlations between left dlPFC beta power and self-reported arousal

To examine the functional significance of left dorsolateral prefrontal cortex (L dlPFC) beta modulation observed during the Reappraise condition, we correlated mean beta power (15–30 Hz) extracted from this ROI with self-reported arousal across conditions. Spearman tests (*N* = 40) showed a specific negative association under Natural Negative: higher L dlPFC beta related to lower arousal (rho = −0.340, 95% CI [−0.595, −0.022], *p* = 0.032). No other condition yielded reliable effects (Reappraise: rho = −0.011, 95% CI [−0.330, 0.310], *p* = 0.947; Neutral: rho = 0.151, 95% CI [−0.178, 0.450], *p* = 0.352).

## 4 Discussion

The present findings reveal that adult attachment orientations are reflected in distinct patterns of cortical oscillatory dynamics. We interpret these patterns as markers of fronto-parietal mechanisms involved in emotion regulation. Although cognitive reappraisal did not elicit a significant overall theta power increase, individuals with higher attachment anxiety exhibited reduced theta-band activity in the right dorsolateral prefrontal cortex (dlPFC) during reappraisal, suggesting attenuated recruitment of cognitive control mechanisms (Domic-Siede et al., 2024; Ertl et al., 2013; Zhang et al., 2025; Zhao et al., 2021). In contrast, expressive suppression was characterized by decreased beta power in the right parietal cortex and precuneus—regions involved in self-referential attention—consistent with attentional disengagement from self-related processing (Northoff et al., 2006; Ochsner et al., 2012; Spitzer and Haegens, 2017).

During reappraisal, attachment anxiety was linked to lower beta power and attachment avoidance to higher beta power in the left dlPFC. These results indicate that reappraisal and suppression engage distinct oscillatory systems and that attachment orientations modulate their neural implementation. Frontal theta activity indexes top-down control processes during reappraisal, whereas beta oscillations reveal complementary functions: frontal beta reflects regulatory control differences associated with attachment (Abid et al., 2025; Domic-Siede et al., 2025b; Etkin et al., 2015; Feeser et al., 2014; He et al., 2023; Long et al., 2020; Zhao et al., 2021), and parietal beta decreases during suppression suggest reduced self-referential attention (Northoff et al., 2006; Ochsner et al., 2012).

Frontal midline theta is a well-established marker of cognitive control engagement, particularly during emotion regulation (Cavanagh and Frank, 2014), while beta-band oscillations in fronto-parietal networks sustain ongoing cognitive or sensorimotor states by inhibiting change (Engel and Fries, 2010; Spitzer and Haegens, 2017). From this broader perspective, reduced frontal theta in higher attachment anxiety individuals may reflect difficulty in recruiting executive control under emotional stress—consistent with heightened reactivity and low regulatory flexibility (Diamond and Fagundes, 2010; Mikulincer and Shaver, 2019; Silva, 2005). Conversely, enhanced frontal beta in avoidant individuals could indicate rigid top-down control supporting emotional detachment (Knyazev, 2007; Mikulincer and Shaver, 2019). Thus, insecure attachment may map onto extremes of regulatory control: under-engagement in attachment anxiety and over-engagement in attachment avoidance.

Adopting an attachment framework, our oscillatory results provide neural evidence for these distinct regulatory tendencies. Higher attachment anxiety individuals showed reduced right-dlPFC theta during reappraisal, reflecting inefficient recruitment of cognitive control and a “hyperactivating” regulatory style easily overwhelmed by limbic-driven arousal (Domic-Siede et al., 2024; Long et al., 2020; Gillath et al., 2005; Mikulincer and Shaver, 2019; Vrtička and Vuilleumier, 2012). Higher attachment avoidance individuals, in turn, exhibited higher left-dlPFC beta, consistent with a “deactivating” style emphasizing cognitive distancing and suppression (Dörfel et al., 2014; Vrtička and Vuilleumier, 2012; Engel and Fries, 2010; Stoll et al., 2016). Together, these findings reveal opposite deviations in the recruitment of frontal control-related oscillations: Higher attachment anxiety individuals under-engaged theta-mediated control, while higher attachment avoidance individuals over-engaged beta-mediated inhibition. These complementary signatures for “hyperactivating” versus “deactivating” tendencies clarify how attachment orientations correspond to distinct neural mechanisms of regulation (Domic-Siede et al., 2025a, 2025b; Mikulincer and Shaver, 2019).

Bowlby’s concept of internal working models (IWMs) posits that early relationships shape emotion regulation strategies (Bowlby, 1969, 1973, 1982; Cassidy and Shaver, 2016). Our findings provide neurophysiological support for attachment-related individual differences: variation along the dimensions of attachment anxiety and avoidance was associated with distinct neural responses during regulation. An anxious attachment IWM—marked by fear of rejection—tended to coincide with weaker engagement of frontal control, whereas an avoidant attachment IWM—marked by emotional deactivation—tended to be associated with stronger prefrontal control. This aligns with evidence that attachment orientations are biologically embedded in emotion-related brain systems (Vrtička and Vuilleumier, 2012; Long et al., 2020; White et al., 2023).

Consistent with the Social Neuroscience of Attachment (SoNeAt) perspective (Vrtička, 2017; White et al., 2023), attachment orientations corresponded to differential recruitment of oscillatory networks. Behaviorally, higher attachment anxiety individuals tend to hyperactivate emotions, while avoidant individuals rely on suppression and withdrawal (Shaver and Mikulincer, 2007; Mikulincer and Shaver, 2019). Our electrophysiological findings mirror these profiles: lower frontal theta (under-regulation) in higher attachment anxiety and higher frontal beta (over-regulation) in higher attachment avoidance (Mikulincer and Shaver, 2019; Vrtička and Vuilleumier, 2012). Thus, attachment insecurity biases both emotional appraisal and regulation implementation, reflected here in frontal theta under-engagement and frontal beta over-engagement. Integrating biological levels of analysis into attachment theory (Thompson et al., 2022), our findings suggest that early relational experiences leave measurable neurophysiological imprints. Higher attachment anxiety individuals—with internal models of inconsistent support—showed vigilance and reduced top-down control, whereas higher attachment avoidance individuals—with models of self-reliance—showed enhanced inhibitory control and emotional disengagement. These results illustrate how IWMs are instantiated in the brain’s regulatory circuitry.

Moreover, our results align with the Neuro-Anatomical Model of Attachment (NAMA; Long et al., 2020), which proposes that attachment anxiety involves a hyper-reactive limbic system and reduced prefrontal regulation, whereas avoidance entails attenuated emotional appraisal and increased control. The observed reduction in right-dlPFC theta among anxious individuals and elevation of left-dlPFC beta among avoidant individuals fit this model (Klimesch, 2012; Vrtička and Vuilleumier, 2012; Cassidy and Shaver, 2016; Mikulincer and Shaver, 2016), offering empirical support for NAMA’s framework.

Finally, by capturing oscillatory markers of attachment-related regulation, this study bridges behavioral and neurophysiological evidence. Prior electrophysiological work, including ERP research, has also linked attachment anxiety to enhanced sustained processing of negative stimuli (e.g., larger LPP amplitudes; Domic-Siede et al., 2025a). From a complementary perspective, the present source-level oscillatory findings highlight reduced frontal theta engagement during reappraisal among higher-attachment anxiety individuals, consistent with attenuated recruitment of cognitive control. Moreover, recent connectivity analyses have demonstrated that attachment orientations also modulate large-scale synchronization during emotion regulation, with anxious attachment showing reduced fronto-parietal theta coupling and avoidant attachment showing enhanced beta-band connectivity (Domic-Siede et al., 2025b). Together, these results indicate that IWMs are reflected not only in local oscillatory dynamics but also in the coordination of distributed regulatory networks, linking interpersonal dispositions to neural processes and advancing an integrative understanding of attachment and emotion regulation.

### 4.1 Clinical implications

Difficulties in emotion regulation constitute a core transdiagnostic process implicated across a wide range of psychological conditions, including anxiety, depression, personality disorders, and trauma-related syndromes (Aldao et al., 2010; Cludius et al., 2020). From a dimensional perspective, variation in emotion regulation capacity is shaped by stable interpersonal dispositions, among which attachment orientations play a central role (Bowlby, 1969; Mikulincer and Shaver, 2016; Cassidy and Shaver, 2016). Attachment anxiety and attachment avoidance reflect characteristic regulatory tendencies—hyperactivating and deactivating, respectively—that bias how individuals engage cognitive and affective control mechanisms across contexts. In this framework, the present findings indicate that different attachment orientations are associated with distinct neural implementations of emotion regulation, highlighting potential relevance for transdiagnostic, individualized, and preventative intervention approaches.

Frontal theta and prefrontal beta activity appear to index different modes of emotion regulation, converging with long-standing clinical observations that anxiously attached individuals often experience emotional overwhelm, whereas avoidantly attached individuals rely more on detachment or suppression (Shaver and Mikulincer, 2002; Mikulincer and Shaver, 2019). At the neural level, diminished right dlPFC theta during reappraisal suggests that anxious individuals under-engage prefrontal control, which may explain why cognitive strategies like reframing/reappraisal often feel ineffective. In contrast, increased left dlPFC beta in avoidant individuals indicates sustained cognitive control or distancing, consistent with a rigid regulatory style aimed at minimizing affective engagement.

These patterns imply differentiated therapeutic approaches. For individuals with higher attachment anxiety, interventions that strengthen prefrontal regulatory engagement—such as cognitive reappraisal training, mindfulness, or neurofeedback targeting frontal theta—could enhance their capacity for top-down control. Preliminary evidence shows that neurofeedback protocols aimed at increasing frontal theta improve emotional regulation and reduce stress-related reactivity (Kang et al., 2014; Ros et al., 2013). For avoidant clients, therapy may focus on reducing excessive control and fostering safe emotional expression. Psychotherapeutic approaches such Emotionally focused Individual Therapy (Brubacher, 2017) or attachment-based therapies can help them notice their tendency to withdraw and gradually tolerate emotional activation (Fonagy and Bateman, 2006). By reframing high beta activity as a neural marker of overcontrol, therapists can use psychoeducation or biofeedback to promote awareness and relaxation during emotionally charged moments.

These findings highlight that attachment-related regulation patterns have identifiable neural correlates, suggesting that the habitual emotional responses of anxiously and avoidantly attached individuals are instantiated in distinct brain dynamics that can nonetheless be modified through targeted intervention and relational experience. Communicating this to clients can reduce self-blame and enhance engagement: anxious individuals may feel validated knowing their difficulty in calming down has a neural basis that can change with practice, while avoidant individuals may begin to view their detachment as a learned neural habit that can be softened through relational safety.

More broadly, integrating these neural insights into attachment-based interventions could enrich psychoeducation in individual, couple, or group therapy. Understanding that anxious “hyperactivation” and avoidant “deactivation” correspond to distinct neural patterns may help clients and partners move from criticism to self-compassion—recognizing that these reactions are brain-based strategies for emotional protection that can be modified through therapeutic experience.

### 4.2 Limitations and future directions

Several limitations should be noted when interpreting these findings.

First, the present study employed a nested stimulus–condition design, in which each set of negative images was consistently paired with a specific regulation instruction. As a result, stimulus identity was confounded with regulation condition, and analyses based on normative IAPS ratings cannot fully rule out subtle stimulus-specific influences. Accordingly, the present findings should be interpreted as reflecting differences between regulatory contexts implemented over normatively matched stimulus sets, rather than as effects that are entirely independent of stimulus identity. Future studies would benefit from between-participant counterbalancing of stimulus sets across regulation conditions (e.g., using a Latin-square rotation), which would allow for a more direct assessment of stimulus-specific variability while avoiding within-participant carryover effects.

Relatedly, although the negative stimulus sets were closely matched in mean normative arousal values, the present analyses do not allow us to determine whether the full distributions of arousal within each set were equivalent, or whether a subset of higher-arousal images may have disproportionately contributed to observed condition effects. In addition, because baseline arousal ratings were not collected in the present sample, changes in subjective arousal should be interpreted relative to normative values rather than individual pre-regulation baselines. Future studies incorporating item-level baseline ratings or passive viewing conditions would provide a more direct way to assess distributional differences in stimulus arousal and to further reduce potential bias associated with nested designs.

In addition, the number of trials per condition was relatively modest by design (15 trials per condition prior to artifact rejection). After preprocessing, participants retained a high proportion of trials (on average approximately 14–15 trials per condition), with a balanced distribution across conditions. Although this high retention rate supports the reliability of the estimated neural measures, the limited number of trials per condition may constrain the precision of source-level estimates and should be considered when interpreting the findings.

Importantly, we sought to partially address this limitation by conducting an additional trial-level mixed-effects analysis of self-reported arousal, in which stimulus variability was explicitly modeled as a random factor nested within condition. This analysis confirmed that, although stimulus-related variability was present, the main effect of Condition remained robust after accounting for this variance. Moreover, the magnitude of stimulus-level variability was substantially smaller than between-subject variability, and no evidence was found that a small subset of stimuli disproportionately drove the observed effects. These findings suggest that, while the nested design introduces a theoretical source of bias, the behavioral effects observed in the present study are unlikely to be reducible to stimulus-specific influences alone. This provides additional support for the interpretation that the observed neural effects are unlikely to be fully explained by stimulus-specific variability. Nevertheless, because stimulus identity was not counterbalanced across conditions, future studies employing fully crossed or partially counterbalanced designs will be necessary to definitively isolate regulatory effects from stimulus-driven variability.

Beyond these methodological considerations, the present study focused primarily on arousal as an index of regulatory success, based on dimensional models of emotion in which affective valence and emotional intensity are dissociable components (Russell, 1980; Lang et al., 1997). Within this framework, successful regulation may involve changes in emotional intensity (arousal), changes in affective valence (e.g., reductions in experienced unpleasantness), or both, depending on the specific regulatory strategy and context (Gross, 1998, 2015). However, because post-task valence ratings were not collected, the present design does not allow us to determine whether, or to what extent, valence changed in magnitude across regulation conditions. Consequently, potential contributions of valence-related processes to the observed behavioral and neural effects cannot be ruled out and should be considered an important conceptual limitation of the current study.

Future research incorporating trial-by-trial assessments of both arousal and valence will be essential to more fully characterize how different affective dimensions jointly shape emotion regulation and its neural correlates (Cunningham et al., 2004; Cunningham et al., 2013). At the neural level, prior work on cognitive reappraisal suggests that changes in affective valence and evaluative meaning engage medial and ventrolateral prefrontal regions involved in appraisal and semantic reinterpretation (Ochsner et al., 2004; Ochsner and Gross, 2005; McRae et al., 2010, 2012; Wager et al., 2008). Accordingly, future studies could test whether valence-related changes are reflected in prefrontal networks that complement the arousal-related control dynamics observed in oscillatory activity.

Second, the exclusive reliance on EEG constrains spatial precision. Although source reconstruction improves anatomical inference, deep or subcortical structures involved in emotion regulation—such as the amygdala, hippocampus, and limbic regions overall—cannot be accurately localized (Baillet, 2017). More broadly, EEG source reconstruction remains an ill-posed inverse problem, and commonly used methods (including sLORETA) rely on simplifying assumptions such as the quasi-static approximation of Maxwell’s equations, which limits spatial specificity and may lead to overinterpretation of focal cortical sources. Accordingly, the present findings should be interpreted as reflecting distributed fronto-parietal oscillatory patterns rather than precise anatomical localization. Recent work has begun to challenge these assumptions by proposing alternative EEG reconstruction frameworks that explicitly model tissue anisotropy, inhomogeneity, and displacement currents, achieving markedly improved spatial fidelity when validated against simultaneous EEG–fMRI and intracranial recordings (Frank et al., 2025). These approaches represent a promising avenue for future studies seeking to refine the spatial interpretation of oscillatory dynamics. Multimodal approaches combining EEG and fMRI could clarify whether frontal theta activity during reappraisal co-occurs with BOLD activation in prefrontal control regions and reduced limbic reactivity, as observed in prior regulation research (Knyazev, 2012; Wager et al., 2008). Simultaneous EEG–fMRI could therefore strengthen the spatial validity of oscillatory interpretations.

Third, the study’s cross-sectional and correlational design limits causal inference. Attachment orientations and neural activity were measured at a single time point; thus, we cannot determine whether attachment orientations shape neural oscillations or vice versa. Longitudinal and experimental studies—such as attachment security priming (Gillath and Karantzas, 2019)—could examine whether temporary or enduring shifts in attachment security alter theta and beta dynamics during emotion regulation. Demonstrating such causal modulation would substantiate the functional role of attachment in shaping neural control processes.

Fourth, attachment was assessed using the self-report *Experiences in Close Relationships* (ECR) scale (Brennan et al., 1998), which captures conscious attachment tendencies but not deeper representational aspects. Interview-based measures like the *Adult Attachment Interview* (AAI; Main et al., 2008) or narrative tasks may index defensive processes more directly related to emotion regulation (Vrtička and Vuilleumier, 2012). Future studies integrating multiple assessment levels—self-report, representational, and behavioral—would help clarify which components of attachment predict neural variability. For instance, do AAI “dismissing” individuals exhibit the same beta enhancement as self-reported avoidants? Such convergence would strengthen construct validity and rule out method variance.

Conceptually, the paradigm involved regulating emotions elicited by unpleasant but impersonal IAPS images. Although this enhances experimental control, the absence of interpersonal cues means the attachment system may not have been fully engaged; attachment processes are most strongly activated in relational contexts (Mikulincer and Shaver, 2019). Thus, eliciting emotions without interpersonal content could potentially misrepresent or attenuate attachment-related effects. Investigating oscillatory activity during socially or attachment-relevant tasks—such as regulating emotions in response to partner feedback, rejection, or separation cues (Gillath et al., 2005)—would improve ecological validity. Combining neural data with autonomic indices could also reveal whether insecure attachment involves parallel physiological dysregulation (Diamond and Fagundes, 2010).

Cultural and developmental generalizability likewise warrant attention. The current mostly Chilean adult sample limits cross-cultural inference. Emotion regulation and attachment expression vary across societies (Mesquita et al., 2016); in cultures where restraint is normative, even secure individuals might show stronger beta activity during suppression. Comparative studies across individualistic and collectivistic contexts could clarify the cultural embedding of attachment-related neural patterns. Developmentally, longitudinal work tracking oscillatory activity from adolescence into adulthood could reveal when attachment-linked neural differences crystallize (Silvers et al., 2015; McRae et al., 2012). Such work would inform interventions aimed at promoting regulatory flexibility during sensitive periods of brain maturation.

Finally, future research should integrate behavioral, physiological, and computational levels of analysis (Petter et al., 2025). Emotion regulation is multifaceted, involving subjective, expressive, and neural components (Gross, 2015). Linking oscillatory indices with behavioral success (e.g., reductions in subjective arousal or facial expression) would test whether frontal theta predicts regulatory efficacy or beta reflects suppression effort even during reappraisal. Building on work that decodes affect from brain activity and demonstrates cross-modal correspondences (e.g., multivoxel “neural signatures” and ERP–fMRI links; Chang et al., 2015; Bo et al., 2021; Liu et al., 2012; Sabatinelli et al., 2013), and on studies where connectivity and task-evoked signals track regulation success and therapy-related change (Morawetz et al., 2017; Goldin et al., 2013; Dixon et al., 2020), multimodal, machine-learning pipelines are well-positioned to yield composite attachment-regulation profiles with predictive value for vulnerability and treatment responsiveness.

In summary, addressing these methodological and conceptual limitations—through multimodal imaging, longitudinal and experimental designs, diverse samples, and multi-level analyses—will clarify how attachment shapes the brain’s regulatory systems. The present study provides an initial step toward a neurophysiological oscillatory model of attachment-based emotion regulation, paving the way for translational research and individualized interventions.

## 5 Conclusion

Our study demonstrates that adult attachment orientations are reflected in distinct oscillatory dynamics within prefrontal brain networks during emotion regulation. Individuals higher in attachment anxiety exhibited reduced theta-band activity in the right dlPFC during cognitive reappraisal, suggesting diminished engagement of top-down cognitive control. In contrast, those higher in attachment avoidance showed increased beta-band activity in the left dlPFC during reappraisal, indicating a more rigid and cognitively distanced regulatory mode consistent with deactivating strategies (Engel and Fries, 2010; Klimesch, 2012; Vrtička and Vuilleumier, 2012).

These findings bridge attachment theory with neurophysiology, revealing that IWMs described by Bowlby (1969, 1982) have measurable neural correlates when individuals attempt to manage their emotions. The results align with and extend foundational and contemporary frameworks—from Bowlby and Ainsworth’s seminal ideas, through Shaver and Mikulincer’s (2007) hyperactivation/deactivation model, to the SoNeAt (White et al., 2023)—by providing concrete neurodynamic evidence of how attachment insecurity alters emotion regulation at the brain level. Moreover, these neural insights hold translational promise. Frontal theta and prefrontal beta oscillations may serve as potential biomarkers of regulatory capacity in insecurely attached individuals and as targets for interventions aimed at enhancing prefrontal control, such as neurofeedback or brain stimulation (Ros et al., 2013; Kang et al., 2014).

More broadly, this work underscores that attachment is not only a theory of social–emotional behavior but a biologically instantiated process: early relational experiences shape the wiring and oscillatory patterns of neural networks that sustain emotion regulation. By integrating attachment theory with cognitive neuroscience, we obtain a richer understanding of individual differences in emotion regulation—one that bridges interpersonal narratives and the brain’s electrical rhythms.

This integrative perspective invites future research to examine causality, developmental onset, and cultural influences, while encouraging clinicians to consider the neural as well as relational dimensions of emotion-regulation difficulties. Ultimately, fostering attachment security—through supportive relationships or targeted interventions—may yield benefits that are neurobiological as well as psychological, strengthening the brain’s capacity for adaptive emotional control. Prefrontal oscillatory dynamics thus offer a promising window into the interplay between attachment and emotion regulation, with implications for theory, clinical innovation, and wellbeing across the lifespan.

## Data Availability

The raw data supporting the conclusions of this article will be made available by the authors, without undue reservation.

## Footnotes

1
www.biosemi.com

2
http://neuroimage.usc.edu/brainstorm
