# Supplementary material for: Fronto-parietal oscillatory dynamics of emotion regulation as a function of adult attachment orientations
Source: Front Hum Neurosci. 2026 May 7;20:1742378. doi: 10.3389/fnhum.2026.1742378 (PMC13190587; doi:10.3389/fnhum.2026.1742378)
Supplement: Supplementary file 1 [file Data_Sheet_1.docx]

**Supplementary Material**

**Supplementary Table S1. Selected IAPS pictures per condition (Lang et al., 2005)**

|  | Experimental Conditions | | | |
| --- | --- | --- | --- | --- |
| N° | Nat-negative | Suppress | Reappraise | Nat-neutral |
| 1 | 2683 | 2095 | 2130 | 7009 |
| 2 | 2703 | 2205 | 2700 | 7011 |
| 3 | 2710 | 2900 | 2751 | 7012 |
| 4 | 2717 | 6211 | 2811 | 7018 |
| 5 | 3220 | 6231 | 3550 | 7042 |
| 6 | 3230 | 6250 | 6260 | 7045 |
| 7 | 3500 | 6312 | 6350 | 7061 |
| 8 | 6300 | 6560 | 6530 | 2190 |
| 9 | 6315 | 6563 | 6561 | 2215 |
| 10 | 6360 | 6838 | 6562 | 2359 |
| 11 | 6540 | 9041 | 6825 | 2499 |
| 12 | 6550 | 9250 | 6834 | 2518 |
| 13 | 6840 | 9421 | 9050 | 2521 |
| 14 | 9429 | 9427 | 9419 | 2593 |
| 15 | 9900 | 9435 | 9425 | 2594 |

Nat-negative: Natural condition containing negative valence pictures; Nat-neutral: Natural condition containing neutral valence pictures.

**Supplementary Table S2. Descriptive statistics of the selected IAPS pictures per condition.**

|  | Arousal | | Valence | |
| --- | --- | --- | --- | --- |
|  | Mean | SD | Mean | SD |
| Nat-negative | 6.09 | 0.59 | 2.48 | 0.40 |
| Suppress | 5.76 | 0.81 | 2.50 | 0.50 |
| Reappraise | 5.83 | 0.81 | 2.77 | 0.51 |
| Nat-neutral | 3.48 | 0.48 | 5.30 | 0.50 |

Nat-negative: Natural condition containing negative valence pictures; Nat-neutral: Natural condition containing neutral valence pictures. SD = Standard Deviation.

**Supplementary Table S3.** **Multiple comparison test for the arousal and valence of IAPS pictures between conditions**

| **Arousal** | | | | |
| --- | --- | --- | --- | --- |
| Tukey’s test | Mean Diff. | 95.00% CI of diff. | Summary | Adjusted p-value |
| Natural-Neg vs. Suppress | 0.3240 | -0.3415 to 0.9895 | ns | 0.5736 |
| Natural-Neg vs. Reappraise | 0.2633 | -0.4022 to 0.9288 | ns | 0.7222 |
| **Natural-Neg vs. Natural-Neu** | **2.607** | **1.942 to 3.273** | ******** | **<0.0001** |
| Suppress vs. Reappraise | -0.06067 | -0.7262 to 0.6048 | ns | 0.9950 |
| **Suppress vs. Natural-Neu** | **2.283** | **1.618 to 2.949** | ******** | **<0.0001** |
| **Reappraise vs. Natural-Neu** | **2.344** | **1.679 to 3.009** | ******** | **<0.0001** |
|  |  |  |  |  |
| **Valence** | | | | |
| Dunn’s test | Mean rank diff. | | Summary | Adjusted p-value |
| Natural-Neg vs. Suppress | 1.067 | | ns | >0.9999 |
| Natural-Neg vs. Reappraise | -7.667 | | ns | >0.9999 |
| **Natural-Neg vs. Natural-Neu** | **-32.20** | | ******** | **<0.0001** |
| Suppress vs. Reappraise | -8.733 | | ns | >0.9999 |
| **Suppress vs. Natural-Neu** | **-33.27** | | ******** | **<0.0001** |
| **Reappraise vs. Natural-Neu** | **-24.53** | | ******* | **0.0007** |

Natural-neg: Natural condition containing negative valence pictures; Natural-neu: Natural condition containing neutral valence pictures.; ns: not significant.

**Supplementary Table S4. Descriptive statistics** **of self-reported arousal**

| **Condition** | **N** | **Min** | **Max** | **Mean** | **SD** | **SEM** | **95% CI (Lower–Upper)** |
| --- | --- | --- | --- | --- | --- | --- | --- |
| Neutral | 40 | 1.000 | 5.933 | 1.788 | 0.974 | 0.154 | 1.477–2.100 |
| Reappraise | 40 | 1.000 | 5.400 | 2.485 | 1.047 | 0.166 | 2.150–2.820 |
| Suppress | 40 | 1.000 | 6.133 | 2.863 | 1.417 | 0.224 | 2.410–3.317 |
| Negative | 40 | 1.000 | 5.600 | 3.510 | 1.369 | 0.216 | 3.072–3.948 |

**Supplementary Table S5. Shapiro–Wilk test of normality of self-reported arousal**

| **Condition** | **W** | **P value** |
| --- | --- | --- |
| Neutral | 0.7127 | < 0.0001 |
| Reappraise | 0.9534 | 0.0990 |
| Suppress | 0.9317 | 0.0182 |
| Negative | 0.9423 | 0.0412 |

**Supplementary Table S6. Friedman test summary of self-reported arousal**

| **Statistic** | **Value** |
| --- | --- |
| P value | < 0.0001 |
| P value summary | **** |
| Friedman χ²(3) | 57.34 |
| Number of groups | 4 |
| N (subjects) | 40 |

**Supplementary Table S7. Dunn’s multiple comparisons test** **of self-reported arousal**

| **Comparison** | **Rank Sum Diff.** | **Adjusted P** |
| --- | --- | --- |
| Neutral vs. Reappraise | –29.50 | 0.0638 |
| **Neutral vs. Suppress** | –50.50 | < 0.0001 |
| **Neutral vs. Negative** | –82.00 | < 0.0001 |
| Reappraise vs. Suppress | –21.00 | 0.4138 |
| **Reappraise vs. Negative** | –52.50 | < 0.0001 |
| **Suppress vs. Negative** | –31.50 | 0.0382 |

**Supplementary Table S8. Post-hoc pairwise comparisons and effect sizes of self-reported arousal**

| **Comparison** | **p (adjusted)** | **Z** | **r** | **Cohen’s d** | **Magnitude** |
| --- | --- | --- | --- | --- | --- |
| Neutral vs. Reappraise | 0.0638 | — | — | — | < 0.20 Trivial |
| **Neutral vs. Suppress** | < 0.0001 | 3.05 | 0.48 | 0.97 | **Large** |
| **Neutral vs. Negative** | < 0.0001 | 4.96 | 0.78 | 1.57 | **Very large** |
| Reappraise vs. Suppress | 0.4138 | — | — | — | < 0.20 Trivial |
| **Reappraise vs. Negative** | < 0.0001 | 3.18 | 0.50 | 1.00 | **Large** |
| **Suppress vs. Negative** | 0.0382 | 1.91 | 0.30 | 0.60 | **Medium** |

**Supplementary Table S9. Theta band — Main effects and 2-way interactions (ML; N=960; DF=888)**

| **Effect** | **Estimate** | **SE** | **t** | **df** | **p** | **95% CI** |
| --- | --- | --- | --- | --- | --- | --- |
| (Intercept) | 0.028149 | 0.048934 | 0.57523 | 888 | 0.56528 | [-0.067892, 0.12419] |
| Condition_Reappraise | 0.024128 | 0.06593 | 0.36596 | 888 | 0.71448 | [-0.10527, 0.15353] |
| Condition_Suppress | 0.082297 | 0.068769 | 1.1967 | 888 | 0.23174 | [-0.052672, 0.21727] |
| ROI_L ACC | 0.03255 | 0.059136 | 0.55042 | 888 | 0.58217 | [-0.083513, 0.14861] |
| ROI_L dlPFC | 0.036987 | 0.059136 | 0.62545 | 888 | 0.53184 | [-0.079077, 0.15305] |
| ROI_L mPFC | 0.073217 | 0.059136 | 1.2381 | 888 | 0.216 | [-0.042846, 0.18928] |
| ROI_R ACC | 0.080577 | 0.059136 | 1.3626 | 888 | 0.17337 | [-0.035486, 0.19664] |
| ROI_R dlPFC | 0.10691 | 0.059136 | 1.8079 | 888 | 0.070962 | [-0.0091513, 0.22298] |
| ROI_R mPFC | 0.041956 | 0.059136 | 0.70947 | 888 | 0.47822 | [-0.074108, 0.15802] |
| ROI_R vlPFC / IFG | 0.017743 | 0.059136 | 0.30003 | 888 | 0.76422 | [-0.098321, 0.13381] |
| ANX_c | -0.018087 | 0.044787 | -0.40385 | 888 | 0.68642 | [-0.10599, 0.069813] |
| AVD_c | 0.015154 | 0.0413 | 0.36692 | 888 | 0.71377 | [-0.065903, 0.09621] |
| Condition_Reappraise:ROI_L ACC | 0.022228 | 0.082216 | 0.27037 | 888 | 0.78694 | [-0.13913, 0.18359] |
| Condition_Suppress:ROI_L ACC | -0.048065 | 0.082216 | -0.58462 | 888 | 0.55895 | [-0.20942, 0.11329] |
| Condition_Reappraise:ROI_L dlPFC | 0.042631 | 0.082216 | 0.51852 | 888 | 0.60422 | [-0.11873, 0.20399] |
| Condition_Suppress:ROI_L dlPFC | 0.019746 | 0.082216 | 0.24018 | 888 | 0.81025 | [-0.14161, 0.18111] |
| Condition_Reappraise:ROI_L mPFC | -0.004994 | 0.082216 | -0.06074 | 888 | 0.95158 | [-0.16635, 0.15637] |
| Condition_Suppress:ROI_L mPFC | -0.13449 | 0.082216 | -1.6359 | 888 | 0.10222 | [-0.29585, 0.026867] |
| Condition_Reappraise:ROI_R ACC | -0.027419 | 0.082216 | -0.3335 | 888 | 0.73883 | [-0.18878, 0.13394] |
| Condition_Suppress:ROI_R ACC | -0.080605 | 0.082216 | -0.98041 | 888 | 0.32715 | [-0.24196, 0.080755] |
| Condition_Reappraise:ROI_R dlPFC | -0.029588 | 0.082216 | -0.35988 | 888 | 0.71902 | [-0.19095, 0.13177] |
| Condition_Suppress:ROI_R dlPFC | -0.13908 | 0.082216 | -1.6916 | 888 | 0.09107 | [-0.30044, 0.022282] |
| Condition_Reappraise:ROI_R mPFC | 0.059035 | 0.082216 | 0.71805 | 888 | 0.47292 | [-0.10232, 0.22039] |
| Condition_Suppress:ROI_R mPFC | -0.065274 | 0.082216 | -0.79394 | 888 | 0.42744 | [-0.22663, 0.096085] |
| Condition_Reappraise:ROI_R vlPFC / IFG | 0.0053231 | 0.082216 | 0.06475 | 888 | 0.94839 | [-0.15604, 0.16668] |
| Condition_Suppress:ROI_R vlPFC / IFG | -0.058369 | 0.082216 | -0.70995 | 888 | 0.47792 | [-0.21973, 0.10299] |
| Condition_Reappraise:ANX_c | 0.030524 | 0.060342 | 0.50586 | 888 | 0.61308 | [-0.087905, 0.14895] |
| Condition_Suppress:ANX_c | -0.026955 | 0.06294 | -0.42826 | 888 | 0.66856 | [-0.15048, 0.096574] |
| ROI_L ACC:ANX_c | -0.0197 | 0.054124 | -0.36398 | 888 | 0.71596 | [-0.12593, 0.086526] |
| ROI_L dlPFC:ANX_c | -0.056714 | 0.054124 | -1.0478 | 888 | 0.29499 | [-0.16294, 0.049512] |
| ROI_L mPFC:ANX_c | 0.019152 | 0.054124 | 0.35385 | 888 | 0.72354 | [-0.087074, 0.12538] |
| ROI_R ACC:ANX_c | -0.034821 | 0.054124 | -0.64336 | 888 | 0.52016 | [-0.14105, 0.071405] |
| ROI_R dlPFC:ANX_c | 0.040166 | 0.054124 | 0.7421 | 888 | 0.45822 | [-0.06606, 0.14639] |
| ROI_R mPFC:ANX_c | 0.011717 | 0.054124 | 0.21648 | 888 | 0.82866 | [-0.094509, 0.11794] |
| ROI_R vlPFC / IFG:ANX_c | 0.060895 | 0.054124 | 1.1251 | 888 | 0.26085 | [-0.045331, 0.16712] |
| Condition_Reappraise:AVD_c | 0.043305 | 0.055644 | 0.77825 | 888 | 0.43663 | [-0.065904, 0.15251] |
| Condition_Suppress:AVD_c | -0.062006 | 0.05804 | -1.0683 | 888 | 0.28566 | [-0.17592, 0.051906] |
| ROI_L ACC:AVD_c | 0.00094817 | 0.04991 | 0.018997 | 888 | 0.98485 | [-0.097007, 0.098904] |
| ROI_L dlPFC:AVD_c | -0.078228 | 0.04991 | -1.5674 | 888 | 0.11738 | [-0.17618, 0.019727] |
| ROI_L mPFC:AVD_c | -0.018024 | 0.04991 | -0.36112 | 888 | 0.71809 | [-0.11598, 0.079932] |
| ROI_R ACC:AVD_c | 0.03331 | 0.04991 | 0.66739 | 888 | 0.5047 | [-0.064646, 0.13127] |
| ROI_R dlPFC:AVD_c | -0.022212 | 0.04991 | -0.44505 | 888 | 0.6564 | [-0.12017, 0.075743] |
| ROI_R mPFC:AVD_c | -0.0083151 | 0.04991 | -0.1666 | 888 | 0.86772 | [-0.10627, 0.089641] |
| ROI_R vlPFC / IFG:AVD_c | -0.0050048 | 0.04991 | -0.10028 | 888 | 0.92015 | [-0.10296, 0.092951] |

**Supplementary Table S10. Theta band — 3-way interactions**

| **Effect** | **Estimate** | **SE** | **t** | **df** | **p** | **95% CI** |
| --- | --- | --- | --- | --- | --- | --- |
| Condition_Reappraise:ROI_L ACC:ANX_c | -0.098859 | 0.075247 | -1.3138 | 888 | 0.18926 | [-0.24654, 0.048825] |
| Condition_Suppress:ROI_L ACC:ANX_c | -0.028603 | 0.075247 | -0.38012 | 888 | 0.70395 | [-0.17629, 0.11908] |
| Condition_Reappraise:ROI_L dlPFC:ANX_c | 0.015424 | 0.075247 | 0.20498 | 888 | 0.83763 | [-0.13226, 0.16311] |
| Condition_Suppress:ROI_L dlPFC:ANX_c | 0.063355 | 0.075247 | 0.84196 | 888 | 0.40004 | [-0.084328, 0.21104] |
| Condition_Reappraise:ROI_L mPFC:ANX_c | -0.10936 | 0.075247 | -1.4534 | 888 | 0.14647 | [-0.25705, 0.038321] |
| Condition_Suppress:ROI_L mPFC:ANX_c | -0.085503 | 0.075247 | -1.1363 | 888 | 0.25614 | [-0.23319, 0.06218] |
| Condition_Reappraise:ROI_R ACC:ANX_c | -0.056179 | 0.075247 | -0.7466 | 888 | 0.4555 | [-0.20386, 0.091504] |
| Condition_Suppress:ROI_R ACC:ANX_c | -0.0049498 | 0.075247 | -0.06578 | 888 | 0.94757 | [-0.15263, 0.14273] |
| **Condition_Reappraise:ROI_R dlPFC:ANX_c** | **-0.18656** | **0.075247** | **-2.4793** | **888** | **0.013347** | **[-0.33425, -0.03888]** |
| Condition_Suppress:ROI_R dlPFC:ANX_c | -0.013956 | 0.075247 | -0.18547 | 888 | 0.8529 | [-0.16164, 0.13373] |
| Condition_Reappraise:ROI_R mPFC:ANX_c | -0.095767 | 0.075247 | -1.2727 | 888 | 0.20346 | [-0.24345, 0.051916] |
| Condition_Suppress:ROI_R mPFC:ANX_c | -0.075781 | 0.075247 | -1.0071 | 888 | 0.31416 | [-0.22346, 0.071902] |
| Condition_Reappraise:ROI_R vlPFC / IFG:ANX_c | -0.02043 | 0.075247 | -0.27151 | 888 | 0.78606 | [-0.16811, 0.12725] |
| Condition_Suppress:ROI_R vlPFC / IFG:ANX_c | -0.069451 | 0.075247 | -0.92297 | 888 | 0.35627 | [-0.21713, 0.078232] |
| Condition_Reappraise:ROI_L ACC:AVD_c | -0.023202 | 0.069389 | -0.33438 | 888 | 0.73818 | [-0.15939, 0.11298] |
| Condition_Suppress:ROI_L ACC:AVD_c | 0.085975 | 0.069389 | 1.239 | 888 | 0.21566 | [-0.05021, 0.22216] |
| Condition_Reappraise:ROI_L dlPFC:AVD_c | 0.089594 | 0.069389 | 1.2912 | 888 | 0.19697 | [-0.046591, 0.22578] |
| Condition_Suppress:ROI_L dlPFC:AVD_c | 0.10655 | 0.069389 | 1.5356 | 888 | 0.125 | [-0.029633, 0.24274] |
| Condition_Reappraise:ROI_L mPFC:AVD_c | -0.018569 | 0.069389 | -0.2676 | 888 | 0.78907 | [-0.15475, 0.11762] |
| Condition_Suppress:ROI_L mPFC:AVD_c | 0.13878 | 0.069389 | 2.0000 | 888 | **0.045801** | [0.002595, 0.27496] |
| Condition_Reappraise:ROI_R ACC:AVD_c | -0.097556 | 0.069389 | -1.4059 | 888 | 0.1601 | [-0.23374, 0.038629] |
| Condition_Suppress:ROI_R ACC:AVD_c | 0.022813 | 0.069389 | 0.32877 | 888 | 0.74241 | [-0.11337, 0.159] |
| Condition_Reappraise:ROI_R dlPFC:AVD_c | -0.059808 | 0.069389 | -0.86193 | 888 | 0.38896 | [-0.19599, 0.076377] |
| Condition_Suppress:ROI_R dlPFC:AVD_c | 0.037291 | 0.069389 | 0.53742 | 888 | 0.59111 | [-0.098894, 0.17348] |
| Condition_Reappraise:ROI_R mPFC:AVD_c | -0.077131 | 0.069389 | -1.1116 | 888 | 0.26662 | [-0.21332, 0.059054] |
| Condition_Suppress:ROI_R mPFC:AVD_c | 0.13961 | 0.069389 | 2.012 | 888 | **0.044526** | [0.003422, 0.27579] |
| Condition_Reappraise:ROI_R vlPFC / IFG:AVD_c | -0.0839 | 0.069389 | -1.2091 | 888 | 0.22694 | [-0.22008, 0.052285] |
| Condition_Suppress:ROI_R vlPFC / IFG:AVD_c | 0.079416 | 0.069389 | 1.1445 | 888 | 0.25272 | [-0.056769, 0.2156] |

**Supplementary Table S11. Random-effects (SD/correlations; 95% CIs in text)**

| **Band** | **Group** | **Term** | **SD (approx)** | **Key correlations** |
| --- | --- | --- | --- | --- |
| Theta | Subject | Intercept | 0.161 | ρ(Intercept, Reappraise) = −.55; ρ(Intercept, Suppress) = −.77 |
|  |  | Reappraise slope | 0.197 | ρ(Reappraise, Suppress) = .36 |
|  |  | Suppress slope | 0.232 | — |
|  | Subject:ROI | Intercept | 0.048 | — |
| Beta | Subject | Intercept | 0.083 | ρ(Intercept, Reappraise) = −.52; ρ(Intercept, Suppress) = −.56 |
|  |  | Reappraise slope | 0.104 | ρ(Reappraise, Suppress) = .24 |
|  |  | Suppress slope | 0.053 | — |
|  | Subject:ROI | Intercept | 0.031 | — |

**Supplementary Table S12. Beta band — Main effects and 2-way interactions (ML; N=960; DF=888)**

| **Effect** | **Estimate** | **SE** | **t** | **df** | **p** | **95% CI** |
| --- | --- | --- | --- | --- | --- | --- |
| (Intercept) | 0.026168 | 0.036109 | 0.72469 | 888 | 0.46883 | [-0.044701, 0.097036] |
| Condition_Reappraise | 0.050081 | 0.049853 | 1.0046 | 888 | 0.31537 | [-0.047762, 0.14792] |
| Condition_Suppress | 0.033603 | 0.04784 | 0.7024 | 888 | 0.48261 | [-0.06029, 0.1275] |
| ROI_L PL / Precuneus | 0.08056 | 0.047609 | 1.6921 | 888 | 0.090971 | [-0.012878, 0.174] |
| ROI_L dlPFC | -0.029483 | 0.047609 | -0.61927 | 888 | 0.5359 | [-0.12292, 0.063956] |
| ROI_L vlPFC / IFG | 0.019027 | 0.047609 | 0.39965 | 888 | 0.68951 | [-0.074412, 0.11247] |
| ROI_R BA6 | 0.045924 | 0.047609 | 0.9646 | 888 | 0.33501 | [-0.047515, 0.13936] |
| **ROI_R PL / Precuneus** | **0.11308** | **0.047609** | **2.3753** | **888** | **0.017748** | **[0.019645, 0.20652]** |
| ROI_R dlPFC | -0.002915 | 0.047609 | -0.06122 | 888 | 0.9512 | [-0.096353, 0.090524] |
| ROI_R vlPFC / IFG | -0.0096473 | 0.047609 | -0.20264 | 888 | 0.83947 | [-0.10309, 0.083791] |
| ANX_c | -0.021709 | 0.033048 | -0.6569 | 888 | 0.51142 | [-0.086571, 0.043152] |
| AVD_c | -0.0064556 | 0.030475 | -0.21183 | 888 | 0.83229 | [-0.066267, 0.053356] |
| Condition_Reappraise:ROI_L PL / Precuneus | -0.023648 | 0.06659 | -0.35512 | 888 | 0.72258 | [-0.15434, 0.10705] |
| Condition_Suppress:ROI_L PL / Precuneus | -0.066684 | 0.06659 | -1.0014 | 888 | 0.3169 | [-0.19738, 0.064009] |
| Condition_Reappraise:ROI_L dlPFC | 0.061801 | 0.06659 | 0.92808 | 888 | 0.35362 | [-0.068892, 0.19249] |
| Condition_Suppress:ROI_L dlPFC | 0.035532 | 0.06659 | 0.53359 | 888 | 0.59376 | [-0.095161, 0.16623] |
| Condition_Reappraise:ROI_L vlPFC / IFG | -0.054011 | 0.06659 | -0.8111 | 888 | 0.41753 | [-0.1847, 0.076682] |
| Condition_Suppress:ROI_L vlPFC / IFG | -0.033351 | 0.06659 | -0.50083 | 888 | 0.61661 | [-0.16404, 0.097342] |
| Condition_Reappraise:ROI_R BA6 | -0.075097 | 0.06659 | -1.1277 | 888 | 0.25973 | [-0.20579, 0.055596] |
| Condition_Suppress:ROI_R BA6 | -0.045571 | 0.06659 | -0.68435 | 888 | 0.49393 | [-0.17626, 0.085122] |
| Condition_Reappraise:ROI_R PL / Precuneus | -0.059241 | 0.06659 | -0.88964 | 888 | 0.3739 | [-0.18993, 0.071452] |
| **Condition_Suppress:ROI_R PL / Precuneus** | **-0.21613** | **0.06659** | **-3.2457** | **888** | **0.001216** | **[-0.34682, -0.085438]** |
| Condition_Reappraise:ROI_R dlPFC | -0.028492 | 0.06659 | -0.42787 | 888 | 0.66885 | [-0.15919, 0.1022] |
| Condition_Suppress:ROI_R dlPFC | 0.0045054 | 0.06659 | 0.06766 | 888 | 0.94607 | [-0.12619, 0.1352] |
| Condition_Reappraise:ROI_R vlPFC / IFG | -0.027055 | 0.06659 | -0.40629 | 888 | 0.68463 | [-0.15775, 0.10364] |
| Condition_Suppress:ROI_R vlPFC / IFG | -0.053372 | 0.06659 | -0.80149 | 888 | 0.42306 | [-0.18406, 0.077321] |
| Condition_Reappraise:ANX_c | 0.06727 | 0.045627 | 1.4743 | 888 | 0.14075 | [-0.02228, 0.15682] |
| Condition_Suppress:ANX_c | -0.0061149 | 0.043785 | -0.13966 | 888 | 0.88896 | [-0.09205, 0.07982] |
| ROI_L PL / Precuneus:ANX_c | -0.023596 | 0.043573 | -0.54153 | 888 | 0.58828 | [-0.10912, 0.061923] |
| ROI_L dlPFC:ANX_c | 0.024499 | 0.043573 | 0.56225 | 888 | 0.57409 | [-0.06102, 0.11002] |
| ROI_L vlPFC / IFG:ANX_c | 0.056851 | 0.043573 | 1.3047 | 888 | 0.19233 | [-0.028668, 0.14237] |
| ROI_R BA6:ANX_c | -0.018425 | 0.043573 | -0.42286 | 888 | 0.6725 | [-0.10394, 0.067094] |
| ROI_R PL / Precuneus:ANX_c | 0.013448 | 0.043573 | 0.30862 | 888 | 0.75768 | [-0.072071, 0.098967] |
| ROI_R dlPFC:ANX_c | 0.047218 | 0.043573 | 1.0836 | 888 | 0.27882 | [-0.038301, 0.13274] |
| ROI_R vlPFC / IFG:ANX_c | 0.046066 | 0.043573 | 1.0572 | 888 | 0.2907 | [-0.039453, 0.13159] |
| Condition_Reappraise:AVD_c | -0.02609 | 0.042075 | -0.62009 | 888 | 0.53536 | [-0.10867, 0.056488] |
| Condition_Suppress:AVD_c | -0.0075127 | 0.040376 | -0.18607 | 888 | 0.85244 | [-0.086757, 0.071732] |
| ROI_L PL / Precuneus:AVD_c | 0.02595 | 0.040181 | 0.64583 | 888 | 0.51856 | [-0.069879, 0.087843] |
| ROI_L dlPFC:AVD_c | -0.0077429 | 0.040181 | -0.1927 | 888 | 0.84724 | [-0.069969, 0.087753] |
| ROI_L vlPFC / IFG:AVD_c | 0.02439 | 0.040181 | 0.60701 | 888 | 0.544 | [-0.093477, 0.14575] |
| ROI_R BA6:AVD_c | 0.017516 | 0.040181 | 0.43593 | 888 | 0.66299 | [-0.045442, 0.19379] |
| ROI_R PL / Precuneus:AVD_c | -0.020066 | 0.040181 | -0.49938 | 888 | 0.61764 | [-0.24657, -0.007336] |
| ROI_R dlPFC:AVD_c | 0.0089818 | 0.040181 | 0.22353 | 888 | 0.82317 | [-0.14175, 0.097485] |
| ROI_R vlPFC / IFG:AVD_c | 0.0088917 | 0.040181 | 0.22129 | 888 | 0.82492 | [-0.20894, 0.030296] |

**Supplementary Table S13. Beta band — 3-way interactions**

| **Effect** | **Estimate** | **SE** | **t** | **df** | **p** | **95% CI** |
| --- | --- | --- | --- | --- | --- | --- |
| Condition_Reappraise:ROI_L PL / Precuneus:ANX_c | 0.026138 | 0.060946 | 0.42887 | 888 | 0.66812 | [-0.15835, 0.080881] |
| Condition_Suppress:ROI_L PL / Precuneus:ANX_c | 0.074174 | 0.060946 | 1.217 | 888 | 0.22391 | [-0.16933, 0.069905] |
| **Condition_Reappraise:ROI_L dlPFC:ANX_c** | **-0.12695** | **0.060946** | **-2.083** | **888** | **0.037537** | **[-0.22601, -0.013225]** |
| Condition_Suppress:ROI_L dlPFC:ANX_c | -0.022131 | 0.060946 | -0.36313 | 888 | 0.7166 | [-0.11245, 0.12678] |
| Condition_Reappraise:ROI_L vlPFC / IFG:ANX_c | -0.08932 | 0.060946 | -1.4655 | 888 | 0.14313 | [-0.18423, 0.054998] |
| Condition_Suppress:ROI_L vlPFC / IFG:ANX_c | -0.038735 | 0.060946 | -0.63555 | 888 | 0.52523 | [-0.1436, 0.095633] |
| Condition_Reappraise:ROI_R BA6:ANX_c | -0.049711 | 0.060946 | -0.81565 | 888 | 0.41492 | [-0.19713, 0.0421] |
| Condition_Suppress:ROI_R BA6:ANX_c | 0.037916 | 0.060946 | 0.62213 | 888 | 0.53402 | [-0.12378, 0.11545] |
| Condition_Reappraise:ROI_R PL / Precuneus:ANX_c | -0.10639 | 0.060946 | -1.7456 | 888 | 0.08122 | [-0.215, 0.00222]* |
| Condition_Suppress:ROI_R PL / Precuneus:ANX_c | 0.0071684 | 0.060946 | 0.11762 | 888 | 0.9064 | [-0.11577, 0.10483] |
| Condition_Reappraise:ROI_R dlPFC:ANX_c | -0.064618 | 0.060946 | -1.0602 | 888 | 0.28932 | [-0.15549, 0.065117] |
| Condition_Suppress:ROI_R dlPFC:ANX_c | -0.023983 | 0.060946 | -0.3935 | 888 | 0.69404 | [-0.11577, 0.067804]† |
| Condition_Reappraise:ROI_R vlPFC / IFG:ANX_c | -0.077516 | 0.060946 | -1.2719 | 888 | 0.20375 | [-0.15243, 0.06818] |
| Condition_Suppress:ROI_R vlPFC / IFG:ANX_c | -0.004165 | 0.060946 | -0.06834 | 888 | 0.94553 | [-0.11526, 0.10534] |
| Condition_Reappraise:ROI_L PL / Precuneus:AVD_c | -0.00547 | 0.056201 | -0.09733 | 888 | 0.92249 | [-0.16212, 0.058486] |
| Condition_Suppress:ROI_L PL / Precuneus:AVD_c | -0.045186 | 0.056201 | -0.804 | 888 | 0.42161 | [-0.12362, 0.096987] |
| **Condition_Reappraise:ROI_L dlPFC:AVD_c** | **0.12438** | **0.056201** | **2.2132** | **888** | **0.027139** | **[0.01408, 0.23469]** |
| Condition_Suppress:ROI_L dlPFC:AVD_c | -0.042123 | 0.056201 | -0.7495 | 888 | 0.45375 | [-0.15243, 0.06818] |
| Condition_Reappraise:ROI_L vlPFC / IFG:AVD_c | -0.0049584 | 0.056201 | -0.08823 | 888 | 0.92972 | [-0.11526, 0.10534] |
| Condition_Suppress:ROI_L vlPFC / IFG:AVD_c | -0.051817 | 0.056201 | -0.92198 | 888 | 0.35679 | [-0.16212, 0.058486] |
| Condition_Reappraise:ROI_R BA6:AVD_c | -0.013316 | 0.056201 | -0.23693 | 888 | 0.81277 | [-0.12362, 0.096987] |
| Condition_Suppress:ROI_R BA6:AVD_c | -0.075895 | 0.056201 | -1.3504 | 888 | 0.17723 | [-0.1862, 0.034408] |
| Condition_Reappraise:ROI_R PL / Precuneus:AVD_c | 0.06338 | 0.056201 | 1.1277 | 888 | 0.25974 | [-0.046923, 0.17368] |
| Condition_Suppress:ROI_R PL / Precuneus:AVD_c | 0.047897 | 0.056201 | 0.85223 | 888 | 0.39431 | [-0.062406, 0.1582] |
| Condition_Reappraise:ROI_R dlPFC:AVD_c | 0.01758 | 0.056201 | 0.3128 | 888 | 0.75451 | [-0.092723, 0.12788] |
| Condition_Suppress:ROI_R dlPFC:AVD_c | -0.010764 | 0.056201 | -0.19153 | 888 | 0.84815 | [-0.12107, 0.099538] |
| Condition_Reappraise:ROI_R vlPFC / IFG:AVD_c | -0.006393 | 0.056201 | -0.11375 | 888 | 0.90946 | [-0.1167, 0.10391] |
| Condition_Suppress:ROI_R vlPFC / IFG:AVD_c | 0.015964 | 0.056201 | 0.28405 | 888 | 0.77644 | [-0.094339, 0.12627] |

**Supplementary Figures**

**Supplementary Figure S1.**

**Supplementary Figure S1.** **Normalized beta-band (15–30 Hz) power in the right parietal/precuneus (R PL/Precuneus) region across emotion regulation conditions.** Bars represent mean normalized power spectral density (PSD) values (A–B)/(A+B) relative to baseline for Neutral, Reappraise, Suppress, and Negative conditions (N = 40). Beta activity showed a clear decrease during **Suppress** (M = –0.04, SD = 0.21), in contrast to overall positive modulation during **Neutral** (M = 0.07, SD = 0.29), **Reappraise** (M = 0.13, SD = 0.23), and **Negative** (M = 0.14, SD = 0.26) conditions. Error bars denote ±1 SEM. This pattern illustrates the task-related beta desynchronization specific to suppression, consistent with reduced parietal readiness and visuo-attentional engagement during expressive inhibition.
